# SZOA: An Improved Synergistic Zebra Optimization Algorithm for Microgrid Scheduling and Management

**DOI:** 10.3390/biomimetics10100664

**Published:** 2025-10-01

**Authors:** Lihong Cao, Qi Wei

**Affiliations:** 1 School of Management, Guangzhou College of Technology and Business, Guangzhou 510850, China; caolihong@gzgs.edu.cn; 2 Business College, City University of Macau, Macau 999078, China; 3 Department of Economics, University of Southampton, Highfield, Southampton SO17 1BJ, UK

**Keywords:** microgrid scheduling, economic cost optimization, innovation management, Zebra Optimization Algorithm, global optimization

## Abstract

To address the challenge of coordinating economic cost control and low-carbon objectives in microgrid scheduling, while overcoming the performance limitations of the traditional Zebra Optimization Algorithm (ZOA) in complex problems, this paper proposes a Synergistic Zebra Optimization Algorithm (SZOA) and integrates it with innovative management concepts to enhance the microgrid scheduling process. The SZOA incorporates three core strategies: a multi-population cooperative search mechanism to strengthen global exploration, a vertical crossover–mutation strategy to meet high-dimensional scheduling requirements, and a leader-guided boundary control strategy to ensure variable feasibility. These strategies not only improve algorithmic performance but also provide technical support for innovative management in microgrid scheduling. Extensive experiments on the CEC2017 (d = 30) and CEC2022 (d = 10, 20) benchmark sets demonstrate that the SZOA achieves higher optimization accuracy and stability compared with those of nine state-of-the-art algorithms, including IAGWO and EWOA. Friedman tests further confirm its superiority, with the best average rankings of 1.20 for CEC2017 and 1.08/1.25 for CEC2022 (d = 10, 20). To validate practical applicability, the SZOA is applied to grid-connected microgrid scheduling, where the system model integrates renewable energy sources such as photovoltaic (PV) generation and wind turbines (WT); controllable sources including fuel cells (FC), microturbines (MT), and gas engines (GS); a battery (BT) storage unit; and the main grid. The optimization problem is formulated as a bi-objective model minimizing both economic costs—including fuel, operation, pollutant treatment, main-grid interactions, and imbalance penalties—and carbon emissions, subject to constraints on generation limits and storage state-of-charge safety ranges. Simulation results based on typical daily data from Guangdong, China, show that the optimized microgrid achieves a minimum operating cost of USD 5165.96, an average cost of USD 6853.07, and a standard deviation of only USD 448.53, consistently outperforming all comparison algorithms across economic indicators. Meanwhile, the SZOA dynamically coordinates power outputs: during the daytime, it maximizes PV utilization (with peak output near 35 kW) and WT contribution (30–40 kW), while reducing reliance on fossil-based units such as FC and MT; at night, BT discharges (−20 to −30 kW) to cover load deficits, thereby lowering fossil fuel consumption and pollutant emissions. Overall, the SZOA effectively realizes the synergy of “economic efficiency and low-carbon operation”, offering a reliable and practical technical solution for innovative management and sustainable operation of microgrid scheduling.

## 1. Introduction

In the context of intensifying global warming and the deepening implementation of the “dual-carbon” strategy, the low-carbon transition of energy systems has become a core strategic direction for countries pursuing sustainable development [1]. As a key infrastructure that integrates distributed renewable energy, enhances energy efficiency, and reduces carbon emissions, microgrids play a crucial role in this transition. Innovations and applications in microgrid scheduling not only determine the stability and economic viability of energy systems but also directly influence the pace of achieving low-carbon development goals [2,3,4]. From an economic perspective, as the penetration of intermittent renewable energy sources such as photovoltaic (PV) generation and wind turbines (WT) continues to increase in microgrids, the limitations of traditional scheduling models have become evident. On the one hand, the stochastic nature of renewable energy output often leads to supply–demand imbalances, requiring frequent power exchanges with the main grid or activation of controllable units such as fuel cells (FC) and microturbines (MT) to maintain system balance. This not only increases electricity purchase and fuel procurement costs but may also trigger penalty mechanisms due to power fluctuations, further raising operational expenses. On the other hand, the coupled operation of multiple energy sources in microgrids—including renewable energy, controllable sources, and battery storage (BT)—lacks refined scheduling strategies. Issues such as improper timing of storage charging/discharging and coarse allocation of controllable unit output frequently occur, resulting in persistently high pollutant treatment costs, which undermine the economic feasibility of microgrid operations and hinder their large-scale commercialization [5,6,7].

From the low-carbon development perspective, the core objective of microgrid scheduling has shifted from single “cost minimization” to a coordinated optimization of “economic efficiency and low-carbon goals.” Traditional microgrid scheduling that overly relies on fossil-fuel-based controllable sources not only consumes large amounts of coal, natural gas, and other nonrenewable resources but also generates SO_2_, NO_x_, and CO_2_ emissions, which contradict global low-carbon transition trends. Thus, a pressing challenge is designing advanced scheduling strategies that maximize renewable energy utilization and minimize fossil fuel consumption, thereby achieving simultaneous reductions in carbon emissions and operating costs while ensuring supply–demand balance [8,9,10]. In essence, microgrid scheduling involves dynamically allocating the output of various energy sources within a 24 h horizon under multiple constraints, such as generation limits, state-of-charge (SOC) boundaries of storage, and interaction limits with the main grid. Its complexity lies in the characteristics of “multi-variable coupling, high stochasticity, and multi-objective trade-offs”. For example, during daytime PV peaks, it is necessary to coordinate storage charging and reduce controllable unit output, whereas at night, when PV generation is absent, the challenge shifts to balancing storage discharging and grid electricity purchases, while avoiding risks of power imbalance. These requirements place stringent demands on scheduling algorithms, particularly in terms of global exploration, high-dimensional problem-solving capability, and solution stability [11,12,13].

For both reliability and management considerations, the most effective approach to integrating renewable energy into power systems is through the adoption of microgrid structures. Microgrids are often regarded as intelligent distribution systems because their internal management systems must not only ensure supply–demand balance but also formulate optimal operation strategies to minimize energy costs [14,15]. A renewable-energy-based microgrid, defined as a microgrid in which at least one renewable source serves as the primary power supply, has emerged as a focal point of global research. Within this context, optimal scheduling is a critical task of the microgrid Energy Management System (EMS), aiming to minimize operating costs while mitigating load disturbances. To achieve the optimal operating points of various microgrid components, intelligent scheduling strategies must be developed to determine the most efficient operation plan over the scheduling horizon. Optimal scheduling (OS) encompasses decisions on power generation from internal sources, power exchange between the microgrid and the main grid, and charging/discharging schedules of storage units [16,17].

In addressing the microgrid scheduling problem, conventional optimization techniques such as linear programming and dynamic programming provide satisfactory accuracy under deterministic conditions. However, when confronted with the uncertainties of renewable energy output and the coupling constraints among multiple sources, these methods often suffer from discrepancies between model assumptions and real-world scenarios, leading to local optima and failing to satisfy the requirements of “economic–low-carbon” coordinated scheduling. In recent years, swarm intelligence algorithms have attracted significant attention due to their global search capabilities inspired by collective behaviors in nature [18,19]. By mimicking biological group dynamics—such as ant colonies in foraging or bird flocks in migration—these algorithms exploit information exchange and iterative updates among individuals to progressively approach the global optimum. As a result, they exhibit remarkable advantages in solving high-dimensional, nonlinear, and multi-constrained optimization problems [20].

In recent years, an increasing number of swarm intelligence algorithms have been developed. For example, Particle Swarm Optimization (PSO), inspired by the foraging behavior of bird flocks, was introduced by Eberhart and Kennedy [21]; the Genetic Algorithm (GA), based on Darwin’s theory of evolution, was proposed by Holland [5]; and Simulated Annealing (SA), derived from the annealing process in metallurgy, was presented by N. Metropolis et al. [22]. Other notable examples include the Grey Wolf Optimizer (GWO), inspired by the social hierarchy and hunting behavior of grey wolves [23]; Harris Hawks Optimization (HHO), based on the cooperative predation strategy of hawks hunting rabbits [24]; the Dung Beetle Optimizer (DBO), simulating rolling, dancing, foraging, stealing, and reproduction behaviors of dung beetles [25]; the Secretary Bird Optimization Algorithm (SBOA), inspired by the survival behaviors of secretary birds [26]; the Golden Eagle Optimizer (GEO), derived from golden eagles’ speed-regulating hunting behaviors [27]; the Crested Porcupine Optimizer (CPO), simulating defensive behaviors of crested porcupines [28]; the Black Widow Optimization Algorithm (BWO), based on the unique reproductive behavior of black widow spiders [29]; the Northern Goshawk Optimizer (NGO), inspired by the hunting strategies of northern goshawks [30]; the Marine Predators Algorithm (MPA), based on the foraging behavior of marine predators [31]; the Whale Optimization Algorithm (WOA), simulating the hunting strategy of humpback whales with three phases—search, shrinking encircling, and spiral updating [32]; the Snake Optimizer (SO), inspired by the mating behavior of snakes [33]; the Red-Billed Blue Magpie Optimizer (RBMO), derived from the searching, chasing, attacking, and food-storing behaviors of magpies [34]; the Quantum Avian Navigation Optimizer (QANA), inspired by the remarkable navigation abilities of migratory birds [35]; and the Golden Jackal Optimization Algorithm (GJO), which models the cooperative hunting behaviors of golden jackals [36]. Although these algorithms have demonstrated effectiveness in solving specific optimization problems, most of them still suffer from common shortcomings, such as a tendency to fall into local optima and slow convergence speed.

The Zebra Optimization Algorithm (ZOA) [37], as a recently developed swarm intelligence method, constructs its search mechanism by simulating zebras’ foraging behavior (exploration phase) and anti-predation defense strategies (exploitation phase). While it has shown certain potential in solving low-dimensional numerical optimization problems, its standard version exhibits three major limitations when applied to high-dimensional, highly constrained engineering problems such as microgrid scheduling. First, its search mechanism is overly simplistic: during the foraging phase, the algorithm relies solely on the “pioneer zebra” (i.e., the global best individual) to guide the search, with little information exchange among individuals of different fitness levels. This narrow search perspective reduces diversity and hinders exploration. Second, it suffers from insufficient dimensional coordination, as individuals update each decision variable independently without considering interdimensional correlations. Third, its boundary-handling approach is overly coarse, directly truncating infeasible solutions that exceed constraints [38,39,40,41]. These shortcomings not only limit the optimization accuracy of scheduling schemes but also result in higher operating costs and less effective carbon emission reduction, making it difficult to meet the practical demands of microgrid operation for both economic efficiency and environmental sustainability.

The selection of the Zebra Optimization Algorithm (ZOA) as the basis for developing the SZOA is driven by its inherent advantages and targeted adaptability to microgrid scheduling needs. First, the ZOA’s biologically inspired mechanism—simulating zebras’ foraging (exploration) and anti-predation (exploitation) behaviors—naturally balances global search and local refinement, which aligns with the “multi-variable coupling, high stochasticity” characteristics of microgrid scheduling, unlike the behavior of some MAs (e.g., PSO, GA) prone to premature convergence. Second, the ZOA displays low computational complexity without excessive parameter tuning or complex operators, ensuring that subsequent enhancements (e.g., multi-population search) do not introduce prohibitive overhead, suitable for 24 h high-dimensional scheduling.

To address these issues, this paper proposes a Synergistic Zebra Optimization Algorithm (SZOA) and applies it innovatively to microgrid optimal scheduling, aiming to achieve the dual objectives of “reducing economic costs” and “lowering carbon emissions.” The SZOA introduces three core enhancement strategies to overcome the limitations of the standard algorithm. First, a multi-population cooperative search mechanism is designed, which integrates differential vectors formed from the global best, suboptimal, worst, and randomly selected individuals. Learning factors are dynamically assigned based on Euclidean distance, thereby constructing comprehensive search directions, enhancing population diversity, and fully exploiting the potential for maximizing renewable energy utilization and reducing fossil fuel consumption. Second, a vertical crossover–mutation strategy reorganizes positions across different dimensions within each individual and introduces random perturbations. This breaks the limitation of “dimension-wise independent updates”, enabling coordinated optimization of multi-source power outputs in 24 h microgrid scheduling, alleviating local optima in high-dimensional scenarios, and improving the refinement of scheduling solutions. Third, a leader-guided boundary control strategy replaces conventional fixed-boundary truncation. By constructing dynamic constraint intervals centered on the best individual, it ensures the continuity and rationality of decision variables such as storage state-of-charge (SOC) and power outputs, thereby reducing power imbalance risks and penalty costs.

The main contributions of this study are as follows:

(1) A Synergistic Zebra Optimization Algorithm (SZOA) is proposed by integrating a multi-population cooperative search mechanism, a vertical crossover–mutation strategy, and a leader-guided boundary control strategy. These enhancements effectively address the limitations of the standard Zebra Optimization Algorithm (ZOA), namely its narrow search perspective, insufficient dimensional coordination, and coarse boundary handling, thereby improving its performance on complex optimization tasks.
(2) Comprehensive comparative experiments are conducted on benchmark test suites CEC2017 (d = 30) and CEC2022 (d = 10, 20), where the SZOA is evaluated against nine peer algorithms including IAGWO, EWOA, and VPPSO. Statistical analyses using the Wilcoxon rank-sum test and the Friedman mean-rank test confirm the superior optimization accuracy, convergence speed, and robustness of the SZOA.
(3) An “economic–low-carbon” bi-objective microgrid scheduling model is constructed, and the SZOA is successfully applied to the optimal scheduling of a grid-connected microgrid. Simulation results based on a typical day dataset from Guangdong Province demonstrate its practical value in reducing operating costs (with a minimum cost of USD 5165.96) and mitigating carbon emissions.

The remainder of this paper is organized as follows. Section 2 describes the principles of the ZOA and the design details of the SZOA, including the initialization, exploration, and exploitation phases of the ZOA, as well as the mathematical models and pseudocode of the three enhancement strategies. Section 3 presents numerical experiments, parameter settings, population diversity analysis, exploration–exploitation balance analysis, and performance comparisons. Section 4 applies the SZOA to microgrid scheduling, covering the modeling of distributed generation units, objective functions, and constraints, followed by detailed scheduling results. Section 5 concludes the study, highlights its limitations, and discusses directions for future research.

## 2. Zebra Optimization Algorithm and the Proposed Methodology

### 2.1. Zebra Optimization Algorithm (ZOA)

#### 2.1.1. Initialization

The values of the decision variables are determined by the positions of zebras in the search space. Each zebra can be modeled as a member of the Zebra Optimization Algorithm (ZOA), where its elements represent the values of problem variables. A matrix can be used to mathematically model the number of zebras, and the initial positions of zebras in the search space are randomly assigned. Similar to other heuristic algorithms, the ZOA generates a set of candidate solutions randomly within the search space, as shown in Equation (1) [37].

(1) $$X={\left[\begin{array}{c} X_{1}\\ X_{2}\\ \vdots \\ X_{i}\\ \vdots \\ X_{n} \end{array}\right]}_{n\times d}={\left[\begin{array}{cc} \begin{array}{ccc} x_{1,1} & x_{1,2} & \ldots \\ x_{2,1} & x_{2,2} & \ldots \\ \vdots & \vdots & \ddots \end{array} & \begin{array}{ccc} x_{1,j} & \ldots & x_{1,d}\\ x_{2,j} & \ldots & x_{2,d}\\ \vdots & \ddots & \vdots \end{array}\\ \begin{array}{ccc} x_{i,1} & x_{i,2} & \ldots \\ \vdots & \vdots & \ddots \\ x_{n,1} & x_{n,2} & \ldots \end{array} & \begin{array}{ccc} x_{i,j} & \ldots & x_{i,d}\\ \vdots & \ddots & \vdots \\ x_{n,j} & \ldots & x_{n,d} \end{array} \end{array}\right]}_{n\times d}$$

where X denotes the zebra population, $X_{i,j}$ represents the set of variable values of the $i^{th}$ zebra, $x_{i,j}$ denotes the position of the $j^{th}$ decision variable of the $i^{th}$ zebra, n is the population size, and d is the dimensionality of the problem.

In the optimization space, the zebra population is initialized randomly, and their initial positions are determined according to Equation (2):

(2) $$X_{i,j}=\left({ub}_{j}-{lb}_{j}\right)\times rand+{lb}_{j}$$

where $X_{i,j}$ denotes the position of the $j^{th}$ decision variable of the $i^{th}$ zebra, ${ub}_{j}$ and ${lb}_{j}$ represent the upper and lower bounds of the $j^{th}$ variable, respectively, and rand is a uniformly distributed random number within the range $\left(0,1\right)$.

#### 2.1.2. Exploration (Foraging Behavior)

In the early stages, zebras primarily feed on grasses and sedges. However, when their preferred food sources are scarce, they may also consume shoots, berries, bark, roots, and fallen leaves. Zebras typically spend 60–80% of their time grazing, depending on the quality and abundance of vegetation. One subspecies, known as the plains zebra, is considered a pioneering grazer: by consuming the upper layers and canopy of low-nutrient grasses, it creates space for other herbivores that depend on shorter and more nutritious grasses [37]. In the Zebra Optimization Algorithm (ZOA), the best-performing individual in the population is regarded as the pioneer zebra, which guides the other members toward promising regions of the search space. Accordingly, the position update of zebras during the foraging phase can be modeled by Equations (3) and (4):

(3) $$X_{i,j}\left(t+1\right)=X_{i,j}\left(t\right)+rand\times \left({PZ}_{j}\left(t\right)-I\times X_{i,j}\left(t\right)\right)$$

(4) $$X_{i,j}\left(t+1\right)=\left\{\begin{array}{c} X_{i,j}\left(t+1\right),\;\;\;\;\;\;\;\;\;if\;f\left(X\left(t+1\right)\right)<f\left(X\left(t\right)\right)\\ X_{i,j}\left(t\right),\;\;\;\;\;\;\;\;\;\;\;\;\;\;\;\;\;\;\;\;\;\;\;\;\;\;\;\;\;\;\;\;\;\;\;\;\;\;\;\;\;\;\;\;\;\;\;\;\;\;\;\;\;\;else \end{array}\right.$$

where $X_{i,j}\left(t+1\right)$ and $X_{i,j}\left(t\right)$ denote the positions of the current and previous iterations, respectively, rand is a random number uniformly distributed in [0, 1]; I is a randomly selected integer from the set $\left\{1,2\right\}$; ${PZ}_{j}\left(t\right)$ represents the position of the pioneer zebra (i.e., the best solution obtained so far); and $f\left(X\left(t+1\right)\right)$ and $f\left(X\left(t\right)\right)$ are the fitness values of the updated and previous solutions, respectively.

#### 2.1.3. Exploitation (Defense Strategies Against Predators)

During this phase, the primary predator of zebras is lions, but they are also threatened by cheetahs, leopards, wild dogs, brown hyenas, crocodiles, and spotted dogs. Zebra defense strategies vary depending on the predator. Against large predators such as lions and cheetahs, zebras employ evasive maneuvers, including zigzag runing and random lateral movements. In contrast, when facing smaller predators such as wildebeests or hares, zebras adopt a more aggressive approach, grouping together to confuse and intimidate the predator. In the design of ZOA, it is assumed that one of the following two situations occurs with equal probability [37]:

(1) A lion attacks the zebra, prompting an escape response;
(2) Other predators attack the zebra, leading it to adopt an aggressive defense strategy.

In the first case, when a zebra is threatened by a lion, it seeks refuge near its current position. This behavior is modeled by pattern $S_{1}$ in Equation (5). In the second case, when other predators attack, the remaining zebras in the group move toward the targeted zebra and attempt to intimidate and confuse the predator by forming defensive structures. This strategy is represented by pattern $S_{2}$ in Equation (5). The position update of zebras follows the principle that new positions are accepted only if they improve the fitness value, as shown in Equation (6) [37].

(5) $$X_{i,j}\left(t+1\right)=\left\{\begin{array}{c} S_{1}:X_{i,j}\left(t\right)+R\times \left(2\times rand-1\right)\times \left(1-t/T\right)\times X_{i,j}\left(t\right),\;\;\;\;\;\;if\;P_{S}<0.5\\ S_{2}:X_{i,j}\left(t\right)+rand\times \left({AZ}_{j}\left(t\right)-I\times X_{i,j}\left(t\right)\right),\;\;\;\;\;\;\;\;\;\;\;\;\;\;\;\;\;\;\;\;\;\;\;\;\;\;\;\;\;\;\;\;\;\;\;\;else \end{array}\right.$$

(6) $$X_{i,j}\left(t+1\right)=\left\{\begin{array}{c} X_{i,j}\left(t+1\right),\;\;\;\;\;\;\;\;if\;f\left(X\left(t+1\right)\right)<f\left(X\left(t\right)\right)\\ X_{i,j}\left(t\right),\;\;\;\;\;\;\;\;\;\;\;\;\;\;\;\;\;\;\;\;\;\;\;\;\;\;\;\;\;\;\;\;\;\;\;\;\;\;\;\;\;\;\;\;\;\;\;\;\;\;\;\;else \end{array}\right.$$

where t is the current iteration, T is the maximum number of iterations, R is a constant (0.01), $P_{S}$ is a randomly generated number in [0, 1] representing the probability of switching between the two strategies, rand is a random number in [0, 1], and ${AZ}_{j}\left(t\right)$ denotes the position of the zebra under attack.

### 2.2. Proposed Synergistic Zebra Optimization Algorithm (SZOA)

#### 2.2.1. Multi-Population Synergistic Search Mechanism

In the standard ZOA, during the foraging phase, individuals move toward the global best solution PZ using a simple random weight (Equation (3)). This mechanism lacks information exchange among population members, resulting in a narrow search perspective, premature convergence, and susceptibility to local optima. To overcome these limitations, this study proposes a multi-population cooperative search mechanism.

**Figure 1:**
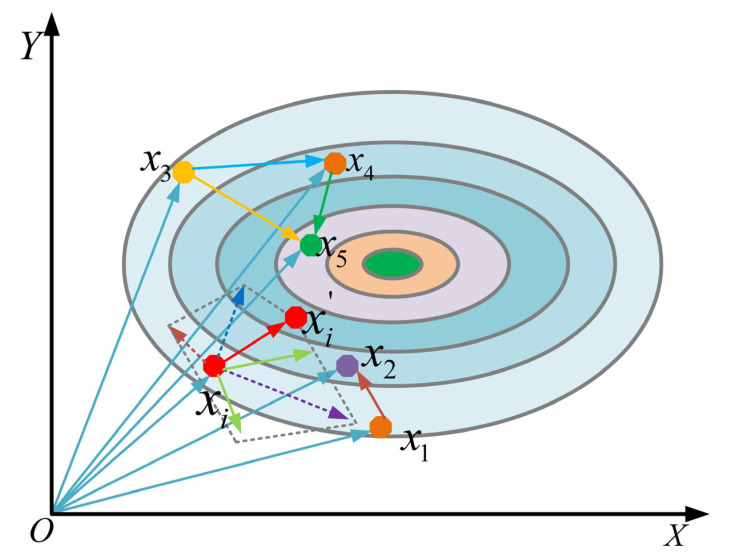
The schematic diagram of the multi-population synergistic search mechanism.

As illustrated in Figure 1, this strategy abandons single-leader guidance and instead constructs a more comprehensive search direction by integrating information from individuals of different fitness levels within the population. Specifically, for each individual $X_{i}$, a guiding vector KA is computed through a weighted sum of multiple differential vectors before its random movement. The position update of the new candidate solution is redefined as

(7) $$X_{i,j}\left(t+1\right)=X_{i,j}\left(t\right)+KA(t)$$

where the guiding vector KA is not generated by a single leader, but is collaboratively composed of four differential vectors, each representing a distinct search direction inspired by interactions within the multi-population framework:

(8) $$KA=\left({LF}_{1}\cdot SF\cdot {Gap}_{1}\right)+\left({LF}_{2}\cdot SF\cdot {Gap}_{2}\right)+\left({LF}_{3}\cdot SF\cdot {Gap}_{3}\right)+\left({LF}_{4}\cdot SF\cdot {Gap}_{4}\right)$$

The four differential vectors are defined as follows:

(9) Gap1=Xbest−Xbetter   Gap2=Xbest−Xworst      Gap3=Xbetter−Xworst  Gap4=Xr1−Xr2            

where $X_{best}$,$\;X_{better}$, and $X_{worst}$ denote the global best, second-best, and worst individuals, respectively, and $X_{r1}\;and\;X_{r2}$ are two randomly selected distinct individuals. The learning factor ${LF}_{k}\left(k=1,2,3,4\right)$ for each vector is dynamically assigned based on its normalized Euclidean distance ${Dist}_{k}$, ensuring that more promising search directions exert greater influence:

(10) $${LF}_{k}=\frac{{Dist}_{k}}{\sum_{k=1}^{4}{Dist}_{k}},\;\;\;k=1,2,3,4$$

Additionally, an adaptive step-size weight SF is introduced to regulate the overall step length:

(11) $$SF=\frac{f(X_{i})}{max\left(f(X_{i})\right)}$$

This design enables individuals with poorer fitness (larger SF) to explore with larger steps, while fitter individuals (smaller SF) perform more refined exploitation. Overall, this strategy significantly enhances intra-population cooperation, balances global exploration and local exploitation, and effectively prevents rapid loss of population diversity.

#### 2.2.2. Vertical Crossover–Mutation Strategy

**Figure 2:**
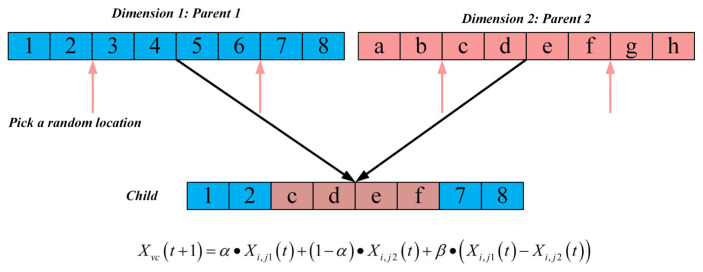
The schematic diagram of vertical crossover–mutation strategy.

In the standard ZOA, population updates rely solely on “global best guidance” and “random perturbations”, lacking the exploitation and recombination of intra-individual dimensional information. This often leads to insufficient dimensional synergy in high-dimensional problems, where some dimensions become trapped in local optima while others retain optimization potential. To address this issue, this study proposes a vertical crossover strategy, as illustrated in Figure 2. By reorganizing the positional information of different dimensions within an individual, this strategy achieves coordinated optimization across dimensions and enhances performance in high-dimensional problems.

The core process of this strategy is as follows: For each individual, two distinct dimensions $\left(j_{1},j_{2}\right)$ are randomly selected. A random weight α~U (0,1) and a perturbation coefficient β~U (−1,1) are then introduced. The two dimensions are recombined via linear combination and perturbation adjustment to generate new dimension values. Subsequently, the recombined individual undergoes boundary checking and fitness evaluation; if the new individual exhibits improved fitness, it replaces the original. The key formula is defined as

(12) $$X_{vc}\left(j\right)=\alpha \cdot X\left(i,j_{1}\right)+\left(1-\alpha \right)\cdot X\left(i,j_{2}\right)+\beta \cdot \left(X\left(i,j_{1}\right)-X\left(i,j_{2}\right)\right)$$

where $X_{vc}$ is the recombined individual, $X\left(i,j_{1}\right)$ and $X\left(i,j_{2}\right)$ denote the positions of the $j_{1}$ and $j_{2}$ dimensions of the current individual, α is the weight coefficient, and β is the perturbation coefficient.

This strategy breaks the limitation of “dimension-independent updates” inherent in traditional algorithms by enabling information complementarity and synergy among dimensions. The weight coefficient α ensures a smooth transition of dimensional information, while the perturbation coefficient β introduces stochastic exploration, effectively preventing the stagnation of evolution caused by partial dimensions being trapped in local optima. Moreover, as this strategy operates solely on intra-individual dimensions, its computational complexity is low, allowing for improved optimization efficiency in high-dimensional problems without increasing the algorithmic burden.

#### 2.2.3. Leader-Based Boundary Control Strategy

In the standard ZOA, the boundary handling method relies on “direct truncation”, which forcibly pulls individuals back into the search space when they exceed the bounds. This approach disrupts the continuity and rationality of individual positions and is particularly prone to creating local optima near the search space boundaries. To address this issue, this study proposes a leader-based boundary control strategy, which uses the global best individual $X_{best}$ as the “guiding center” to construct a dynamic boundary constraint region, enabling smooth boundary regulation of individual positions.

**Figure 3:**
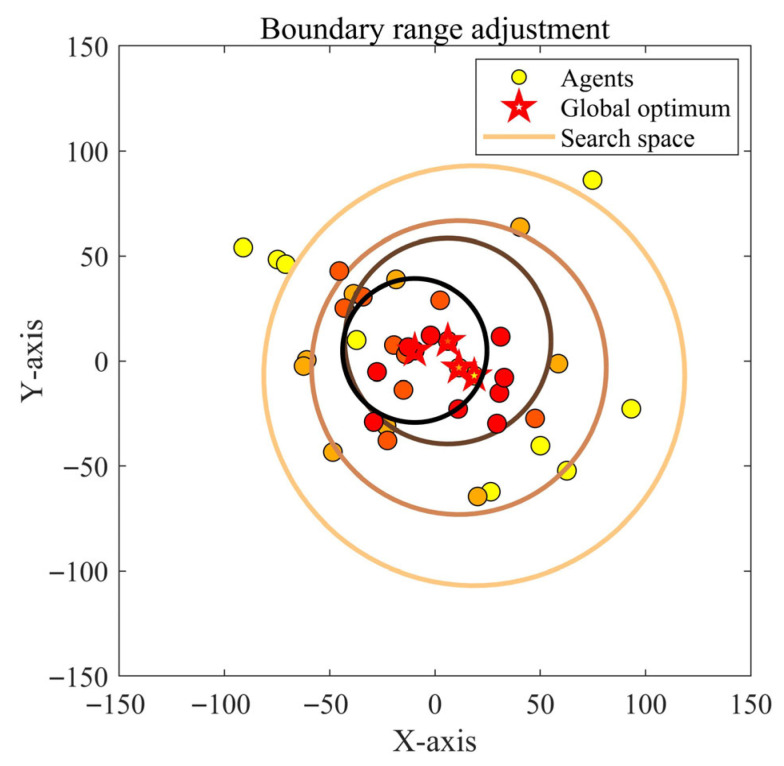
The schematic diagram of the leader-based boundary control strategy.

As illustrated in Figure 3, the core idea of this strategy is to transform the traditional fixed boundaries $\left(lb,\;ub\right)$ into a dynamic constraint range centered on the best individual. Specifically, the lower bound of an individual is defined as the mean of the fixed lower bound and the best individual’s position $\frac{X_{best}\;+\;lb}{2}$, while the upper bound is defined as the mean of the fixed upper bound and the best individual’s position $\frac{X_{best}\;+\;ub}{2}$. Through this dynamic adjustment, individual position updates are always guided toward the “high-quality region” around the global best solution, while avoiding abrupt positional changes caused by direct truncation. The core formula is as follows:

(13) Xit+1=Xbest+ub2Xit+1=Xbest+lb2

Based on the above discussion, the pseudocode for the SZOA is presented in Algorithm 1.
**Algorithm 1.** Pseudo-Code of SZOA.*1:  Initialize Problem Setting (population*$\left(n\right),d$*,*ub,lb*), Max iterations*$\left(T\right)$.*2:  Initialize a set of Zebra’ (*$X_{i}(i=1,2,\ldots n)$*)*.*3:  **while***
t=1:T*** do****4:     *$\left[\sim ,ind\right]=sort\left(f\left(X\right)\right)$*5:     *$X_{best}=X\left(ind\left(1\right),:\right)$*, *$X_{better}=X\left(ind\left(randi\left(\left[2,5\right],1\right)\right),:\right)$*, *$X_{Worst}=X\left(ind\left(randi\left(\left[n-5,n\right],1\right)\right),:\right)$*6:     **for ***i=1:n*7:     **Exploration:****8:        Calculate the fitness *$X\left(t+1\right)$* using Equations (7)–(11).**9:        Update the position of the current individual using Equation (4).**10:       Using Equation (13) for boundary adjustment.**11:    **Exploitation:****12:       Calculate the fitness *$X\left(t+1\right)$* using Equation (5)*.*13:       Update the position of the current individual using Equation (6).**14:       Using Equation (13) for boundary adjustment.**15:    **End for****16:    Update the position of the current individual using Equation (12).**17:    Using Equation (13) for boundary adjustment.**18:    Update the best solution found so far *$X_{best}$.*19:  ****End while****20:  Return *$X_{best}$.

### 2.3. Computational Time Complexity of SZOA

The performance of an algorithm is crucial, but it is equally important to evaluate its time complexity. In many optimization tasks, an algorithm must not only demonstrate excellent performance but also exhibit high real-time efficiency. Time complexity reflects how the algorithm’s runtime scales with the size of the input. Analyzing the time complexity of an optimization algorithm helps estimate its computational cost when handling large-scale problems. In the standard ZOA, the computational complexity of the defined control parameters is O(n×d), where n represents the population size and d denotes the problem dimension. During the initialization phase, the algorithm requires O(n×d) time. Furthermore, over T iterations, the computational complexity for updating individual positions is O(T×n×d). Therefore, the overall computational complexity of the ZOA can be expressed as O(T×n×d). In the proposed SZOA, since only the position update strategy and the objective function evaluation method are improved without introducing additional complexity factors, the time complexity remains O(T×n×d).

## 3. Numerical Experiments

### 3.1. Algorithm Parameter Settings

In this section, the performance of the proposed SZOA is evaluated using the most challenging benchmark suites for numerical optimization, CEC2017 [42] and CEC2022 [43], and then compared with several other algorithms. The comparison algorithms include: Improved Multi-Strategy Adaptive Grey Wolf Optimization (IAGWO) [44], the Enhanced Whale Optimization Algorithm (EWOA) [45], Velocity Pausing Particle Swarm Optimization (VPPSO) [46], improving the search performance of SHADE using linear population size reduction (L-SHADE) [47], Animated Oat Optimization (AOO) [48], the Crested Porcupine Optimizer (CPO) [28], the Dung Beetle Optimization Algorithm (DBO) [49], and the standard Zebra Optimization Algorithm (ZOA) [37]. Parameter settings for all compared algorithms are detailed in Table 1.

### 3.2. Qualitative Analysis of SZOA

#### 3.2.1. Analysis of the Population Diversity

In optimization algorithms, population diversity refers according to the differences among individuals within a population [50,51], with each individual generally representing a candidate solution. A reduction in diversity often results in premature convergence to local optima, which can limit the algorithm’s ability to explore the global search space. Conversely, maintaining greater diversity supports broader exploration of potential solutions and increases the likelihood of identifying the global optimum. In this section, we assess the population diversity of the SZOA approach using Equation (14) [26,52].

(14) $$I_{C}\left(t\right)=\sqrt{\sum_{i=1}^{N}\;\sum_{d=1}^{D}\;{\left(x_{id}\left(t\right)-c_{d}\left(t\right)\right)}^{2}},$$

where $I_{C}\left(t\right)$ denotes the population diversity, N represents the population size, D indicates the problem’s dimensionality, and $x_{id}\left(t\right)$ denotes the value of the i individual in the d dimension at the t iteration. $c_{d}\left(t\right)$ quantifies the dispersion degree of the entire population relative to its center of mass at iteration t, which is calculated using Equation (15).

(15) $$c_{d}\left(t\right)=\frac{1}{D}\sum_{i=1}^{N}\;x_{id}\left(t\right).$$

**Figure 4:**
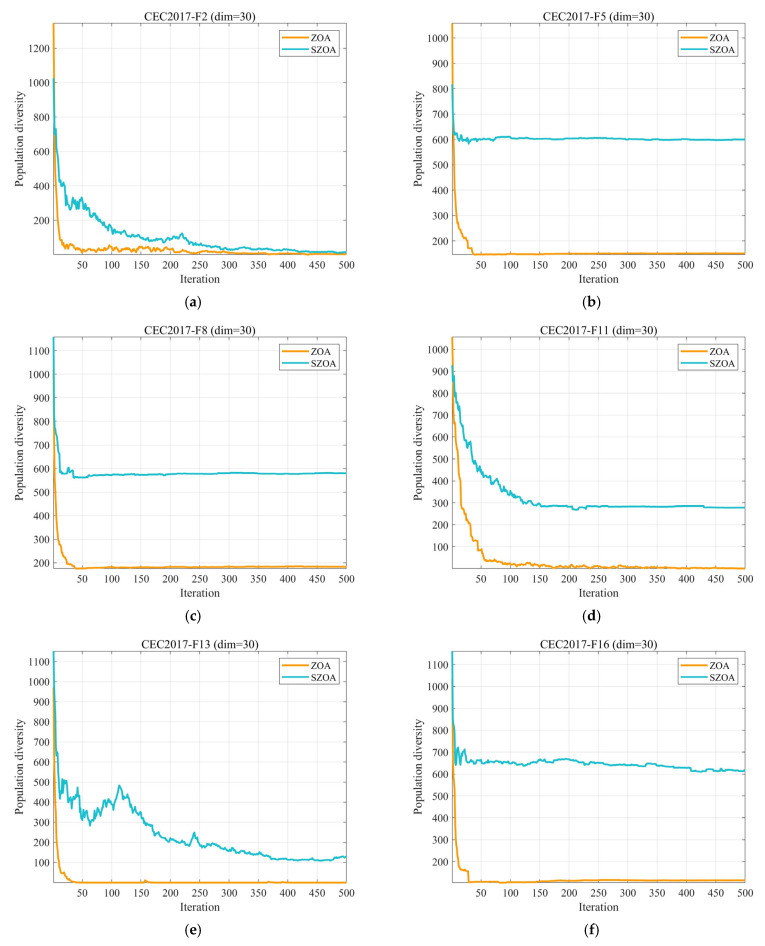

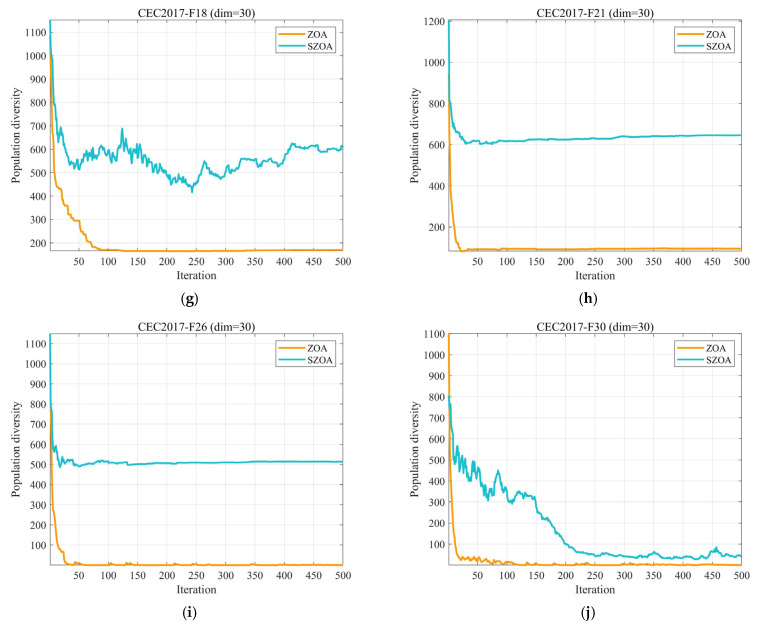
The analysis of the population diversity of SZOA and ZOA.

Figure 4 presents the evolution of population diversity for the SZOA and the original ZOA on the CEC2017 (d = 30) benchmark suite. The results indicate that while the diversity of both algorithms decreases as the iterations progress, the SZOA consistently maintains higher diversity throughout the entire optimization process. For instance, on F18 at iteration 250, the diversity of the ZOA drops below 300, whereas the SZOA maintains it at around 500; on F30 at iteration 400, the SZOA still exhibits diversity above 400, while that for the ZOA falls below 200. This improvement is attributed to SZOA’s multi-population collaborative search mechanism, which integrates differential vectors of individuals with different fitness levels (Gap1–Gap4) and dynamically allocates learning factors, thereby preventing excessive aggregation of individuals. Additionally, the adaptive step size slows down the decay of diversity. In contrast, the ZOA’s single-leader guidance tends to cause rapid diversity loss. This enhanced diversity enables the SZOA to effectively avoid local optima and improve overall optimization performance.

#### 3.2.2. Analysis of the Exploration and Exploitation

In optimization algorithms, both exploration and exploitation play crucial roles. Exploration involves the broad search across different regions of the solution space to discover new areas that may contain the global optimum. Exploitation, on the other hand, focuses on refining and improving existing high-quality solutions through an intensive local search, leveraging current information to achieve higher precision.

Overemphasis on exploration can cause inefficient allocation of computational resources, as the algorithm may scan extensively without sufficiently improving promising solutions, missing opportunities for local refinement. Conversely, excessive exploitation increases the risk of premature convergence to local optima, limiting the search for better solutions in other regions [53,54]. Hence, achieving an appropriate balance between these two processes is essential for algorithmic performance. In this section, we examine the exploratory and exploitative behaviors of the SZOA algorithm, as measured by Equations (16) and (17) [26].

(16) $$Exploration\left(\%\right)=\frac{Div\left(t\right)}{Div_{max}}\times 100\%,$$

(17) $$Exploitation\left(\%\right)=\frac{\left|Div\left(t\right)-Div_{max}\right|}{Div_{max}}\times 100\%,$$

where $Div\left(t\right)$ denotes the measure of diversity at the tth iteration, which is calculated by Equation (18), and $Div_{max}$ denotes the maximum measure of diversity throughout the iteration.

(18) $$Div\left(t\right)=\frac{1}{D}\sum_{d=1}^{D}\frac{1}{N}\sum_{i=1}^{N}|median\left(x_{d}\left(t\right)\right)-x_{id}\left(t\right)|.$$

**Figure 5:**
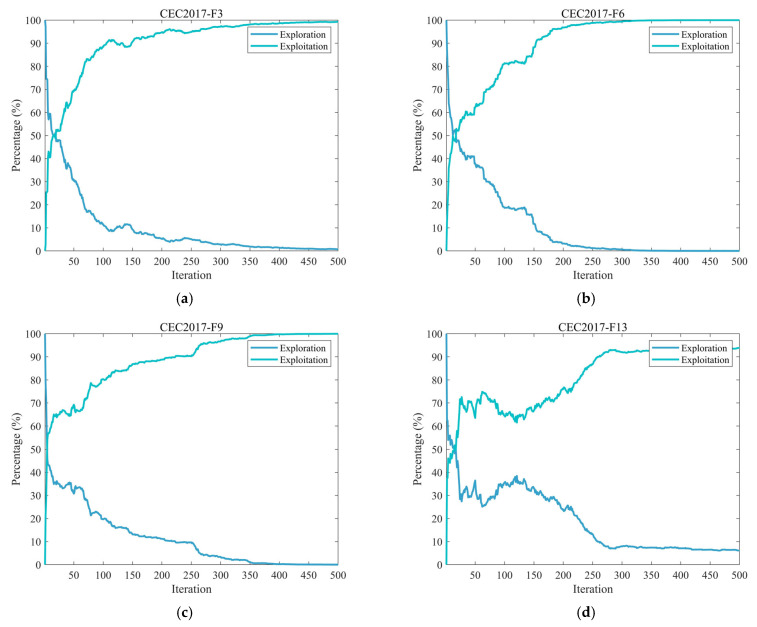

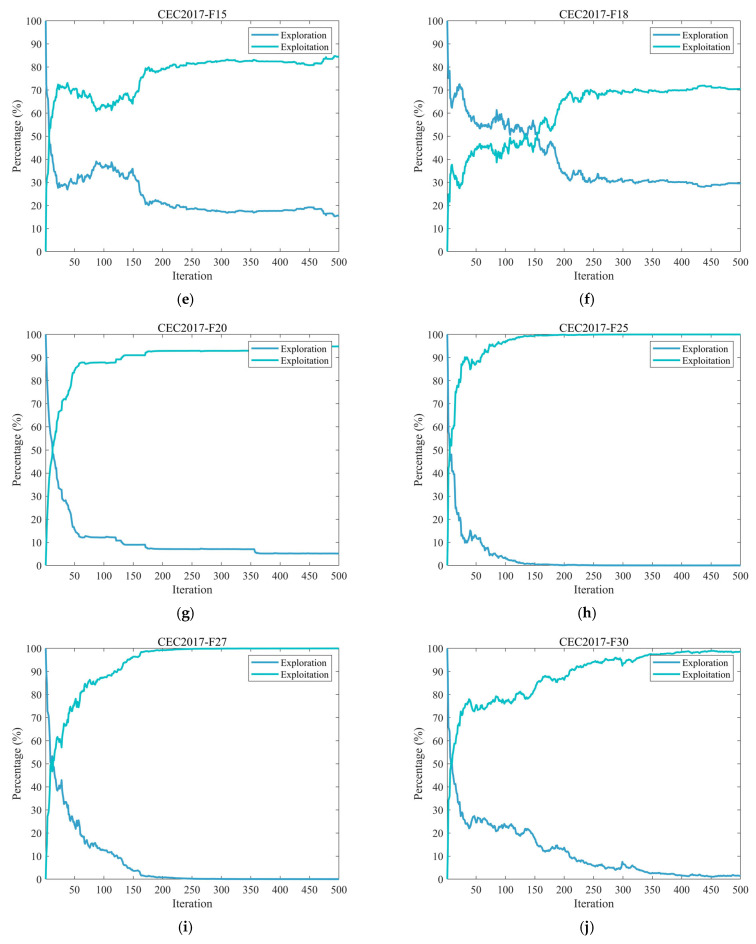
The analysis of the exploration and exploitation of the SZOA.

Figure 5 illustrates the dynamic changes in exploration and exploitation rates of the SZOA on the CEC2017 benchmark functions (all with dimension 30). In the early iterations (0–100), the exploration rate remains between 60% and 80%; for example, on F20 at iteration 50, the exploration rate exceeds 70%, ensuring adequate global search coverage. During the mid-phase (100–300 iterations), the exploration rate decreases to 30–50%, while the exploitation rate rises to 50–70%, achieving a smooth transition from “broad exploration” to “local exploitation”; for instance, on F25 at iteration 200, the exploitation rate reaches 60%. In the late phase (300–500 iterations), the exploitation rate stabilizes at 70–90%, while exploration is retained at 10–30%; for example, on F30 at iteration 500, the exploration rate remains around 20%, balancing solution refinement with avoidance of local optima. This equilibrium is attributed to the SZOA’s design: the multi-population collaborative search mechanism integrates multi-source information to prevent resource wastage and premature convergence, the vertical crossover mutation strategy introduces perturbations to facilitate exploration, and the leader-based dynamic boundary control enhances exploitation efficiency, collectively supporting its superior optimization performance.

#### 3.2.3. Impact Analysis of the Strategy

**Figure 6:**
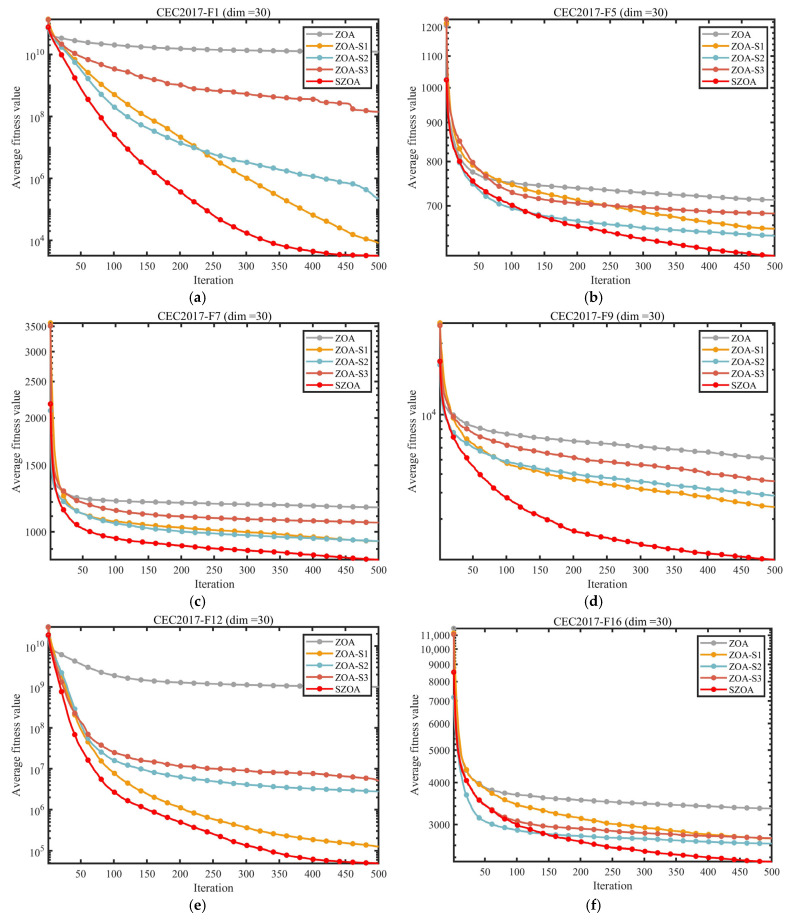

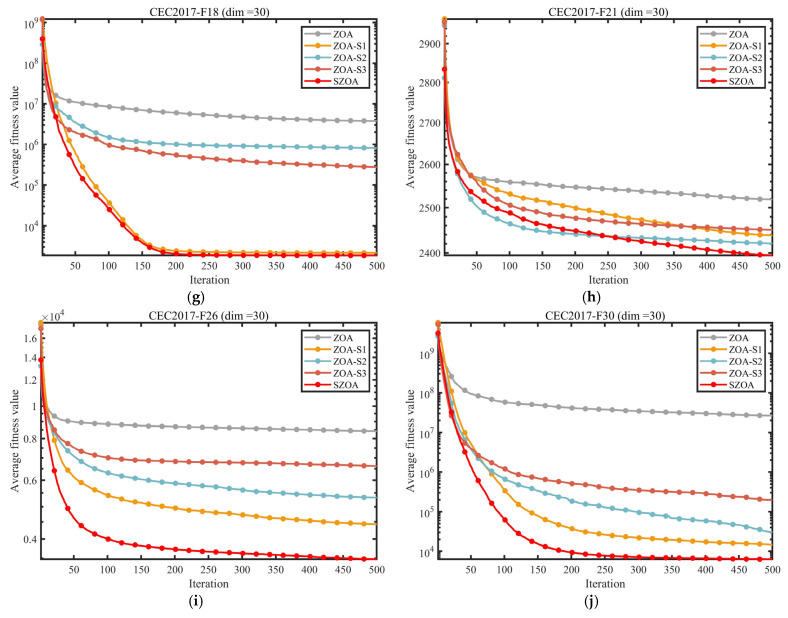
Comparison of different improvement strategies.

**Figure 7:**
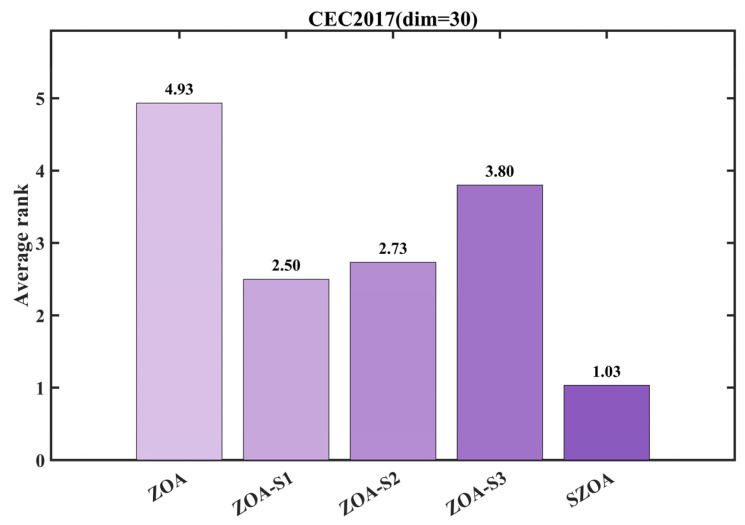
Average ranking of the ZOA improved by different strategies.

To evaluate the individual contributions and synergistic effects of the three enhancement strategies—multi-population synergistic search mechanism (S1), vertical crossover–mutation strategy (S2), and leader-based boundary control strategy (S3)—ablation experiments were conducted using the CEC2017 benchmark suite (dimension d = 30). Five algorithmic variants were designed for comparison: the standard ZOA, ZOA_S1 (incorporating only S1), ZOA_S2 (incorporating only S2), ZOA_S3 (incorporating only S3), and SZOA, which integrates all three strategies. The experimental results are presented in Figure 6 and Figure 7.

The convergence curves in Figure 6 show that the individual strategies contribute differently to ZOA’s performance. ZOA-S1 (incorporating only S1) leverages multi-source individual information, achieving faster convergence than that of the standard ZOA on functions such as F1 and F7, but still underperforming compared to the results for the SZOA. ZOA-S2 (incorporating only S2) optimizes high-dimensional problems through dimension reorganization, performing better than the ZOA on complex functions like F9 and F16, yet struggling to escape local optima. ZOA-S3 (incorporating only S3) enhances stability on functions such as F21 and F30 by reducing abrupt positional changes via dynamic boundary control, but its convergence precision remains limited. In contrast, the SZOA, which integrates all three strategies, demonstrates superior convergence across all benchmark functions; for example, on F18 at iteration 500, the SZOA achieves a significantly lower objective value than that of any single-strategy variant, and on F30, it converges rapidly with minimal fluctuation.

Figure 7 further quantifies these differences through average ranking: the standard ZOA ranks lowest, followed by ZOA-S1, ZOA-S2, and ZOA-S3, while the SZOA leads with an average rank of 2.73, markedly outperforming the other variants whose average ranks all exceed 3.80. These results indicate a significant synergistic effect among the three strategies: S1 provides optimized global search directions, S2 addresses high-dimensional coordination issues, and S3 ensures rational search within boundary regions. The combination not only overcomes the limitations of individual strategies but also enhances the balance between global exploration and local exploitation, enabling the SZOA to surpass both the standard ZOA and its single-strategy variants.

### 3.3. Experimental Results and Analysis of CEC2017 and CEC2022 Test Suite

This section evaluates the performance of the SZOA against other benchmark algorithms on the CEC2017 and CEC2022 test suites, which include four categories of mathematical functions: unimodal, multimodal, composition, and hybrid functions. Multimodal functions, containing multiple local optima, are suitable for assessing the exploration capabilities of new optimizers. Composition and hybrid functions evaluate the algorithms’ ability to avoid local optima, while unimodal functions, containing only a single global optimum, are used to assess exploitation performance.

**Figure 8:**
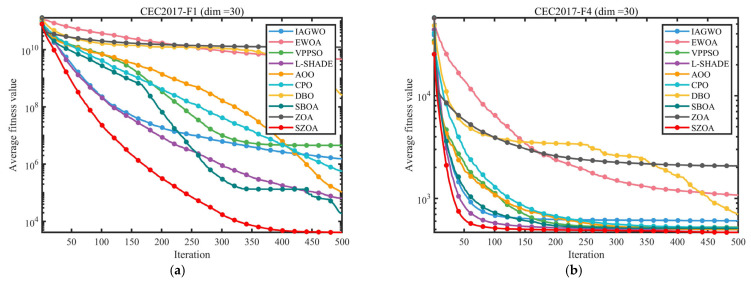

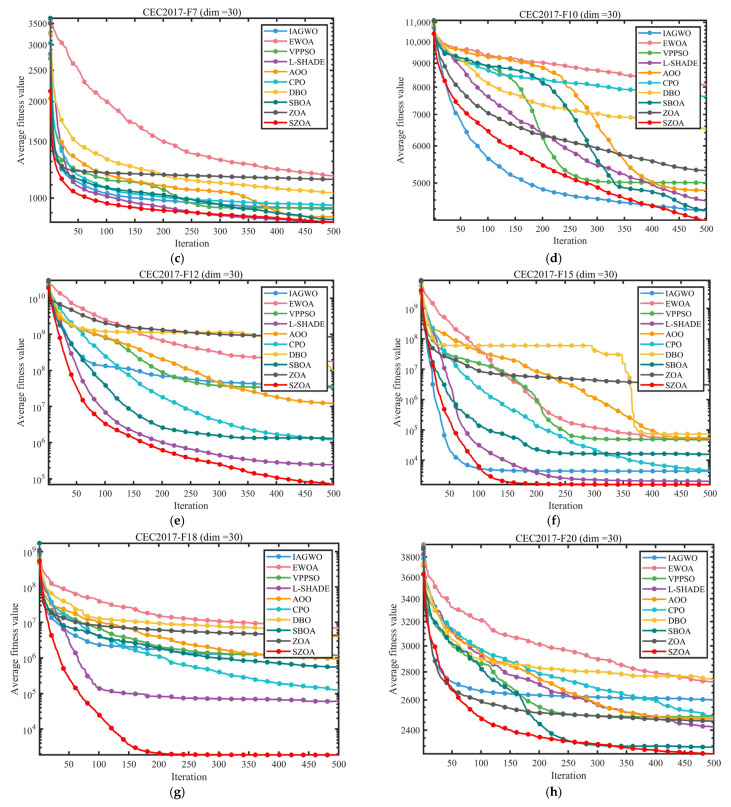

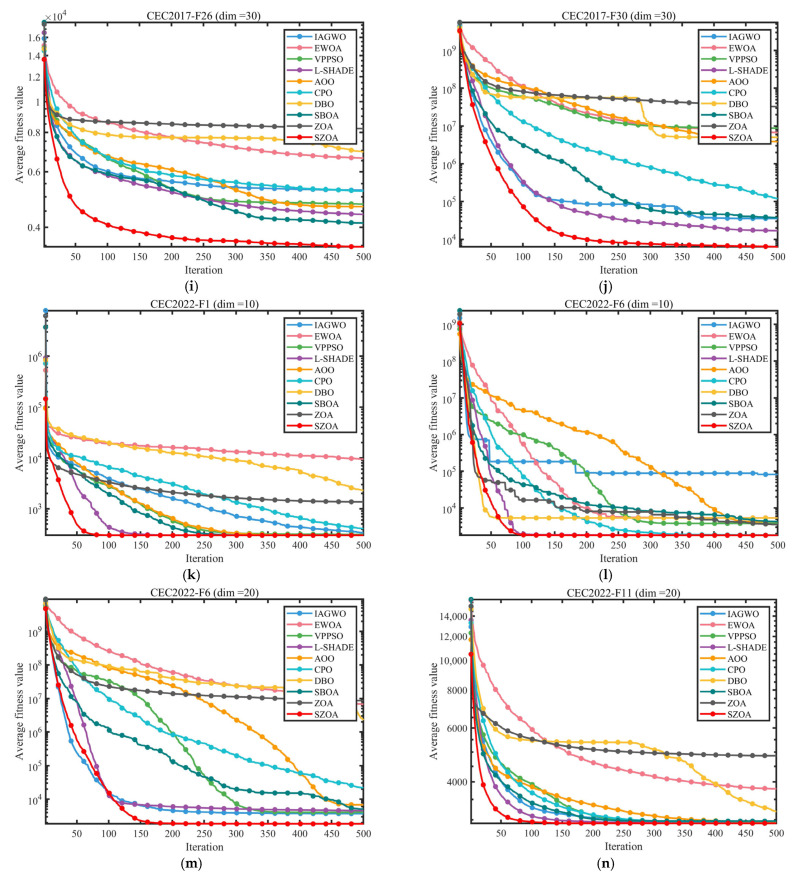
Comparison of convergence speed of different algorithms on CEC2017 and CEC2022 test set.

**Figure 9:**
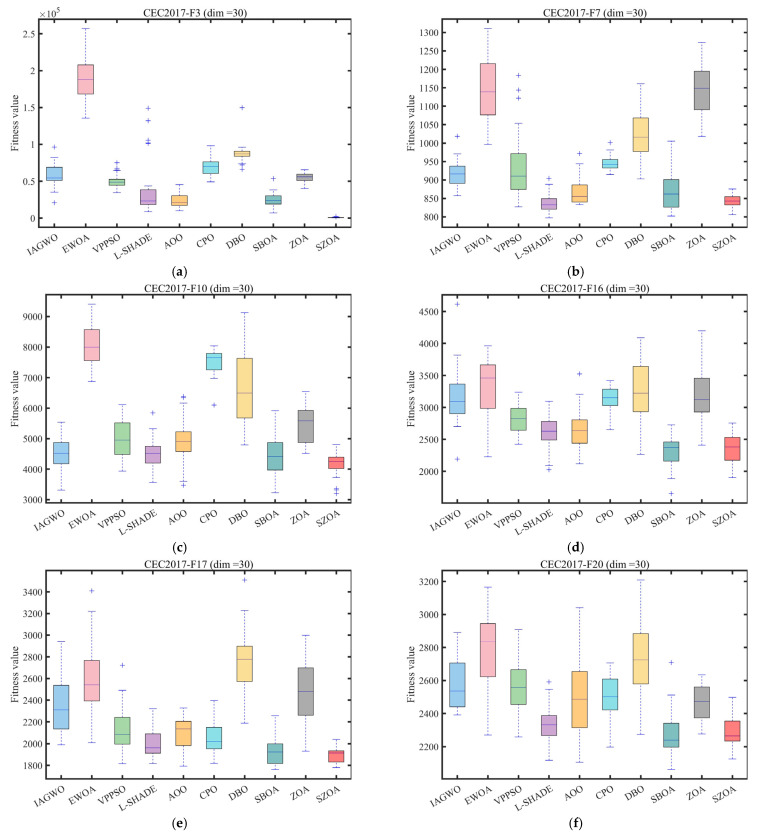

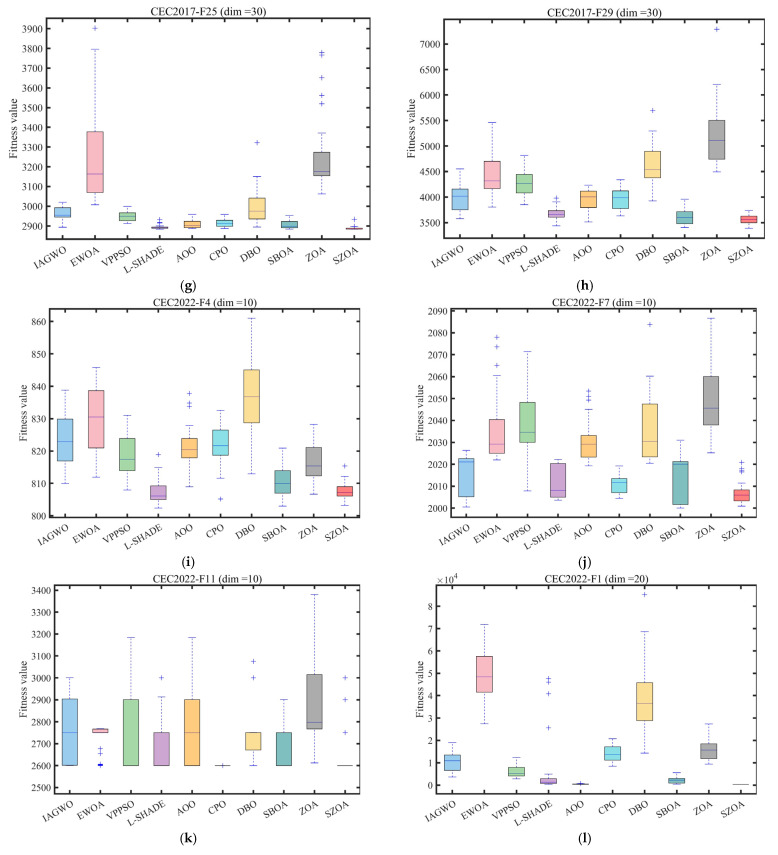

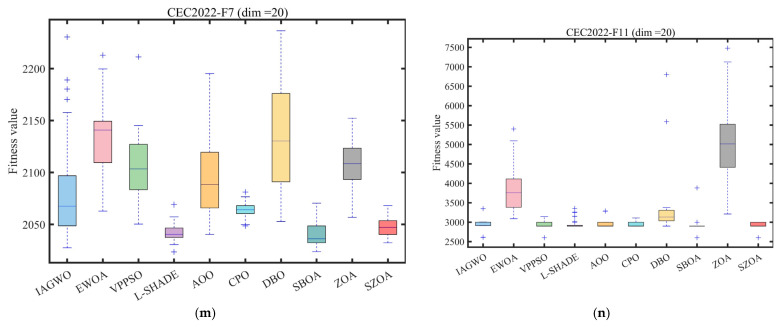
Box plot analysis of different algorithms on the CEC2017 and CEC2022 test set.

To ensure experimental fairness and mitigate randomness, the population size was fixed at 30, the maximum number of iterations was set to 500, and each algorithm was independently run 30 times. The mean (Ave) and standard deviation (Std) of the results were recorded, with the best values highlighted in bold. All experiments were conducted on a Windows 11 system equipped with an AMD Ryzen 7 9700X 8-Core Processor (3.80 GHz), 48 GB of RAM, and MATLAB 2024b. The convergence curves and box plots of the different algorithms are presented in Figure 8 and Figure 9, providing an intuitive visualization of their convergence speed and result distribution characteristics.

Table 2, Table 3 and Table 4 quantitatively compare the optimization performance of the SZOA with nine benchmark algorithms—namely, IAGWO, EWOA, VPPSO, L-SHADE, AOO, CPO, DBO, SBOA, and the original ZOA—on the CEC2017 (dimension) and CEC2022 (dimensions d = 10, d = 20) test suites. The primary evaluation metrics are the mean (Ave) and standard deviation (Std) of the objective function values.

On the CEC2017 test set (d = 30), the SZOA demonstrates clear superiority across the unimodal, multimodal, hybrid, and composition functions. For instance, for the unimodal function F1, which tests local exploitation capability, the SZOA achieves an Ave of 5.2887 × 10^3^, far lower than that of the original ZOA (1.0170 × 10^10^) and also better than that of other high-performing algorithms such as L-SHADE (1.5604 × 10^4^) and SBOA (3.7176 × 10^4^). Its Std is only 6.0862 × 10^2^, indicating strong solution stability. For the multimodal function F30, which evaluates global exploration and avoidance of local optima, the SZOA attains an Ave of 5.8597 × 10^3^—approximately 1/16 of that for the original ZOA (9.7294 × 10^4^)—with an Std of 1.5783 × 10^3^, much lower than the results for DBO (2.4650 × 10^7^) and EWOA (3.3193 × 10^6^), demonstrating stable convergence in complex search spaces.

For the CEC2022 test set, the SZOA’s advantages are further highlighted across different dimensional scenarios. For d = 10, the theoretical optimum of unimodal function F1 is 3.0000 × 10^2^, which the SZOA precisely reaches with an Ave of 3.0000 × 10^2^ and an extraordinarily low Std of 3.1667 × 10^−14^. In contrast, the original ZOA records an Ave of 1.3227 × 10^3^ and an Std of 1.6245 × 10^3^, while the results for IAGWO, EWOA, and other algorithms also exceed 3.0000 × 10^2^, indicating that the SZOA achieves highly accurate optimization for low-dimensional simple functions. For the higher-dimensional scenario (d = 20), the optimization difficulty of F1 significantly increases; the SZOA still maintains an Ave of 3.0000 × 10^2^ with an Std of 2.3065 × 10^−3^, whereas the ZOA and IAGWO record Ave values of 1.6695 × 10^4^ and 1.1055 × 10^4^, respectively. This confirms the effectiveness of the SZOA’s vertical crossover–mutation strategy in addressing “dimension interdependence insufficiency”; by recombining and perturbing internal dimensions, the strategy enables complementary and coordinated information exchange among dimensions, avoiding local stagnation even in high-dimensional spaces.

The convergence curves in Figure 8 provide an intuitive dynamic view of the SZOA’s performance advantage. For representative functions such as F1, F12, F18, and F30 from CEC2017 and F1, F6, and F11 from CEC2022, the SZOA’s curves consistently lie below those of other algorithms, indicating faster convergence. For example, for F12 (a composition function with multiple local optima) of CEC2017, the SZOA quickly reduces the objective function from 10^7^ to 10^5^ within the first 50 iterations, whereas the results for the ZOA and DBO remain at 10^8^–10^9^. By iteration 500, the SZOA achieves 5.3085 × 10^4^, much lower than those for the ZOA (1.0004 × 10^9^) and DBO (1.4215 × 10^8^). This rapid convergence is attributed to the SZOA’s multi-population synergistic search mechanism, which integrates differential vectors of the global best ($X_{best}$), second-best ($X_{better}$), worst ($X_{worst}$), and two random individuals ($X_{r1}$, $X_{r2}$) and dynamically allocates learning factors (${LF}_{k}$) based on Euclidean distances to construct a comprehensive search direction, overcoming the narrow search perspective and slow convergence of the standard ZOA.

Figure 9 shows box plots to quantify result stability. For CEC2017 functions F7, F10, F16, and F25 and for CEC2022 functions F4, F7, and F11, the SZOA exhibits significantly narrower boxes with medians closer to theoretical optima and no obvious outliers. For instance, for F7 of CEC2017, the SZOA’s box ranges from approximately 820 to 860, with a median around 840, while the results for the ZOA range from 900 to 1200, with multiple outliers above 1200. For F4 (d = 10) of CEC2022, the SZOA’s box width is less than 5, whereas the IAGWO and EWOA exhibit widths of approximately 20 and 30, respectively, demonstrating higher consistency across independent L-SHADEs. This high stability results from the SZOA’s leader-based boundary control strategy, which replaces fixed boundaries with dynamic constraint zones centered on the best individual, effectively avoiding abrupt position changes caused by “direct truncation” in the ZOA and reducing local optimum traps near search space boundaries.

Overall, the quantitative results in Table 2, Table 3 and Table 4, combined with the intuitive analyses from Figure 8 and Figure 9, indicate that the synergistic effect of the SZOA’s three core enhancement strategies achieves comprehensive superiority over the original ZOA and other comparison algorithms in terms of optimization accuracy, convergence speed, and result stability. The algorithm demonstrates robust adaptability across different dimensions and function types (unimodal, multimodal, hybrid, and composition), providing solid performance support for subsequent applications in engineering problems such as microgrid optimization scheduling.

### 3.4. Statistical Analysis

Statistical analysis is indispensable for optimizing algorithms, as it provides a framework for researchers to systematically assess and compare the effectiveness of different methods. This process supports informed decision making when identifying the optimal approach for particular research objectives. In this section, the performance of the SZOA algorithm is evaluated through the Wilcoxon rank-sum test and the Friedman test, with comprehensive descriptions of the methodology and outcomes provided.

#### 3.4.1. Wilcoxon Rank-Sum Test

In this subsection, the Wilcoxon rank-sum test [55] is employed to assess whether significant differences exist in the performance of the SZOA algorithm, without relying on assumptions of normality. Compared to the traditional *t*-test, the Wilcoxon test offers greater flexibility, as it remains applicable to data with non-normal distributions or outliers. The test statistic W for the Wilcoxon rank-sum test is defined by Equation (19).

(19) $$W=\sum_{i=1}^{n_{1}}R\left(X_{i}\right),$$

where $R\left(X_{i}\right)$ denotes the rank of $X_{i}$ among all observations. The test statistic U is calculated by Equation (20).

(20) $$U=W-\frac{n_{1}\left(n_{1}+1\right)}{2},$$

For larger sample sizes, U is approximately normally distributed by Equation (21) and Equation (22).

(21) $$\mu_{U}=\frac{n_{1}n_{2}}{2},$$

(22) $$\sigma_{U}=\sqrt{\frac{n_{1}n_{2}\left(n_{1}+n_{2}+1\right)}{12}},$$

and the standardized statistic Z is calculated by Equation (23).

(23) $$Z=\frac{U-\mu_{U}}{\sigma_{U}},$$

A significance level of 0.05 was adopted to determine whether the results of each SZOA run exhibited a statistically significant difference from those of other algorithms. Under the null hypothesis ($H_{0}$), it is assumed that no significant difference exists between the two algorithms. If the p-value is less than 0.05, $H_{0}$ is rejected, indicating a significant performance difference; otherwise, it is retained. The p-value is presented in Appendix A.2

Table 5 quantifies the performance differences between the SZOA and nine benchmark algorithms on the CEC2017 (d = 30) and CEC2022 (d = 10, d = 20) test suites using the Wilcoxon rank-sum test (*p* = 0.05) in the form of “(+/=/-)”, where “+” indicates that SZOA is significantly superior. This test does not require the assumption of normality, allowing for the objective exclusion of random effects. For CEC2017 (d = 30), the SZOA achieves a “+” value of 30 against the ZOA, EWOA, and two other algorithms (i.e., SZOA performs significantly better for all 30 functions), a “+” value of 29 against the VPPSO and L-SHADE (only 1 function shows no advantage), and a “+” value of 27 compared to the IAGWO and AOO (3 functions show no advantage), demonstrating clear superiority in high-dimensional scenarios. For CEC2022, when d = 10, SZOA attains a “+” value of 12 compared to the IAGWO, ZOA, and four other algorithms (all 12 functions are better) and a “+” value of 11 compared to the EWOA and DBO. When d = 20, the results are similar, with only the SBOA showing a “+” value of 9 (3 functions show no advantage). These results indicate that the Wilcoxon test confirms the statistical significance of the SZOA’s performance improvements and demonstrates that its superiority is stable across different dimensionalities, providing critical support for the reliability of its performance.

#### 3.4.2. Friedman Mean Rank Test

In this subsection, the Friedman test [56] is used to determine the overall ranking of the MECOA relative to other methods. As a nonparametric approach, the Friedman test is suitable for comparing median performance differences across three or more matched groups. It is particularly well-suited for repeated measures or block designs, and is often employed as a robust alternative to ANOVA when the assumption of normality is violated. The Friedman test statistic is calculated according to Equation (24).

(24) $$Q=\frac{12}{nk\left(k+1\right)}\sum_{j=1}^{k}R_{j}^{2}-3n\left(k+1\right),$$

where n is the number of blocks, k is the number of groups, and $R_{j}$ is the rank sum for the j-th group. When n and k are large, Q follows approximately a $\chi^{2}$ distribution, with k−1 degrees of freedom.

As shown in Table 6, the performance rankings for the CEC2017 (d = 30) and CEC2022 (d = 10, 20) test suite are presented, where “M.R” denotes the mean rank and “T.R” denotes the total rank. Smaller rank values indicate superior overall algorithm performance. As a nonparametric method, this test does not rely on the assumption of normality and can effectively compare median performance differences among multiple algorithms, making it particularly suitable for the overall evaluation of numerical optimization algorithms.

For CEC2017 (d = 30), the SZOA achieved a mean rank of only 1.20, with a total rank of 1, significantly outperforming the SBOA (mean rank 2.57, 2nd place) and the original ZOA (mean rank 8.80, 9th place). For CEC2022 (d = 20), the SZOA maintained a mean rank of 1.25 and remained in 1st place, while the original ZOA reached a mean rank of 8.83 (total rank 10). Other benchmark algorithms, such as the IAGWO (mean rank 5.75) and EWOA (mean rank 8.58), also lagged far behind the SZOA. These results indicate that the SZOA consistently demonstrates superior comprehensive optimization capability across both high-dimensional, complex test suites and benchmark suites from different years.

**Figure 10:**
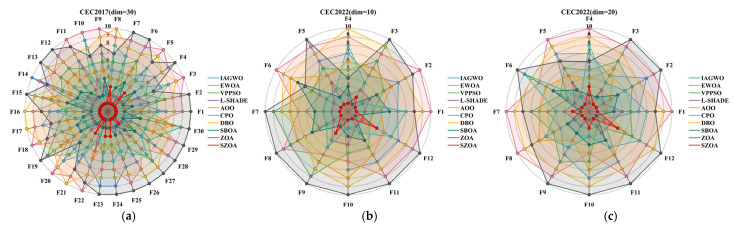
Distribution of rankings of different algorithms.

Figure 10 visualizes the ranking distributions of different algorithms for each function of the CEC2017 (d = 30) and CEC2022 (d = 10, d = 20) test suites, further confirming the statistical results in Table 6. For CEC2017 (d = 30), for representative functions such as F1, F5, F9, and F12, the SZOA consistently ranks 1st, with no function ranked below the top 2. Even for more complex multimodal functions like F26 and F30, the SZOA maintains 1st place, whereas the original ZOA mostly ranks between 8th and 10th, and the IAGWO and EWOA distribute between 3rd and 7th. For CEC2022 (d = 10, d = 20), the SZOA similarly demonstrates concentrated and leading rankings: for d = 10, it ranks 1st for functions such as F1, F3, and F6; for d = 20, although it ranks 2nd for a few functions like F11 and F12, it predominantly holds 1st place. Among the comparison algorithms, only the SBOA and L-SHADE occasionally reach 2nd place, while the rest are mostly ranked 4th or lower.

In summary, the quantitative rankings in Table 6, combined with the visual distributions in Figure 10, indicate that the SZOA’s three synergistic strategies—multi-population collaborative search, vertical crossover–mutation, and leader-based boundary control—enable not only superior performance for individual functions but also consistent overall superiority across different dimensions and function types. The stability and leading position of its rankings further confirm the rationality and effectiveness of the algorithm design.

### 3.5. Runtime Comparison Analysis of SZOA and ZOA

**Figure 11:**
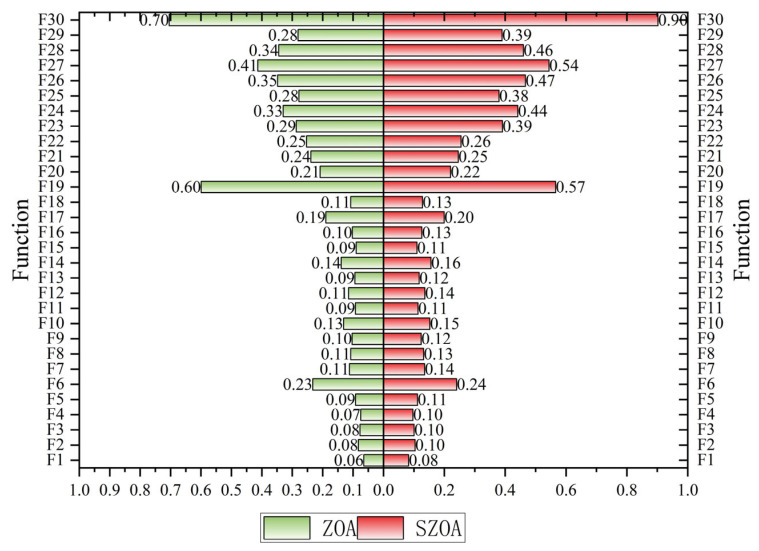
The average runtime of the SZOA and ZOA for the test functions.

Building on the previous research findings, the improved SZOA demonstrates significantly better overall performance compared to that of the standard ZOA. This section focuses on analyzing the computational time cost differences between the two algorithms for the CEC2017 benchmark. To ensure fairness, both the SZOA and ZOA were configured using standardized parameter settings, which are entirely consistent with those used in the preceding sections. The average runtime for each algorithm was calculated based on 30 independent runs. Figure 11 presents the average computation time (in seconds) required by each algorithm to solve the test functions.

From the data distribution in the graph (Figure 11), it can be observed that the running time of the SZOA is slightly higher than that of the original ZOA for most test functions. For instance, for certain functions, the average running time of the SZOA is approximately 0.06–0.70 s, while the results for the SZOA reach 0.08–0.90 s. In a few specific cases (such as F1, where ZOA needs 0.06 s and SZOA requires 0.08 s), the difference in running time becomes more pronounced. However, the overall time cost remains within the same order of magnitude, with no exponential increase.

This slight increase in computation time primarily stems from the three enhanced strategies integrated into the SZOA: the multi-population synergistic search mechanism, the vertical crossover–mutation strategy, and the leader-based boundary control strategy, which introduce additional overhead. Although these new operations add extra computational costs, theoretical time complexity analysis indicates that the SZOA does not introduce higher-order complexity factors. Its time complexity remains consistent with that of the original ZOA, both exhibiting O(T×n×d). Therefore, the difference in running time between the two algorithms remains within an acceptable range.

## 4. Evaluation of the Proposed SZOA for Microgrid Optimal Scheduling

### 4.1. Microgrid Optimal Scheduling Model

The microgrid scheduling system constructed in this study considers a typical day (24 h) scheduling period and incorporates renewable energy sources, conventional controllable generation units, energy storage devices, and interactions with the main grid. The specific configuration is as follows:

(1) **Renewable energy sources**: Photovoltaic (PV) and wind turbine (WT) generation systems, whose outputs depend on natural conditions and are represented using typical day forecast data.
(2) **Conventional controllable generation units**: Fuel cells (FC), micro gas turbines (MT), and small internal combustion engines (GS), whose outputs can be flexibly adjusted according to scheduling requirements.
(3) **Energy storage devices**: Batteries (BT), used to smooth power fluctuations, implement peak shaving and valley filling, and maintain system stability.
(4) **Main grid interaction**: Acts as a supplementary resource for microgrid power balance, allowing both power purchase and sale, with interaction capacity limited by preset upper bounds.

The core parameters of each distributed generation unit are defined in the parameter function in the code, including power limits, operation cost coefficients, and fuel cost coefficients. Detailed parameter values are presented in Table 7, providing the data foundation for model construction.

### 4.2. Mathematical Models of Distributed Generation Units

#### 4.2.1. Renewable Energy Model (PV and WT)

The outputs of PV and WT are constrained by natural conditions. In the code, typical day forecast data are used as fixed inputs. The model expressions are [57,58]

(25) PPV(t)=PPV, pred (t)PWT(t)=PWT, pred (t),

where t is the scheduling time (t=1,2,… ,24);$\;P_{PV}(t)$ and $P_{WT}(t)$ denote the actual outputs of PV and WT at time t (kW); $P_{PV,\;\mathrm{pred}\;}(t)$ and $P_{WT,\;\mathrm{pred}\;}(t)$ denote the forecasted outputs of PV and WT at time t (kW).

#### 4.2.2. Conventional Controllable Generation Model (FC, MT, GS)

FC, MT, and GS are controllable generation units. Their outputs are only constrained by their respective power limits. The model expressions are [57,58]

(26) PFC,min≤PFC(t)≤PFC,maxPMT,min≤PMT(t)≤PMT,maxPGS,min≤PGS(t)≤PGS,max,

where $P_{FC}(t)$, $P_{MT}(t)$, and $P_{GS}(t)$ denote the outputs of FC, MT, and GS at time t (kW); $P_{FC,min}=0\;kW;\;P_{FC,max}=40\;kW;\;P_{MT,min}=0\;kW;\;P_{MT,max}=40\;kW;P_{GS,min}=0\;kW;\;P_{GS,max}=40\;kW$.

#### 4.2.3. Energy Storage System Model (BT)

The BT model includes charge/discharge power constraints and state-of-charge (SOC) constraints. In the code, dynamic control is implemented through the BT function, as follows:

Charge/discharge power constraints:

(27) $$P_{BT,min}\leq P_{BT}(t)\leq P_{BT,max},$$

where $P_{BT}(t)$ is the BT charge/discharge power at time t (kW, positive for charging, negative for discharging); $P_{BT,min}=-40\;kW,\;P_{BT,max}=40\;kW$ are the BT power limits.

SOC constraints and update [6]:

(28) SOCmin≤SOC(t)≤SOCmaxSOC(t+1)=SOC(t)−PBT(t)CBT,

where SOC(t) is the BT state-of-charge at time t; $SOC_{min}=0.2,\;SOC_{max}=0.8$, define the safe SOC range; $C_{BT}=40\;kW$ is the BT capacity; $SOC\left(1\right)=0.4$ is the initial SOC.

#### 4.2.4. Main Grid Interaction Model (GRID)

The GRID acts as a power balance supplement. Its interaction power is limited by preset capacity. The model expression is

(29) $$P_{GRID,min}\leq P_{GRID}(t)\leq P_{GRID,max},$$

where $P_{GRID}(t)$ is the microgrid-to-GRID interaction power at time t (kW, positive for purchasing power, negative for selling power); $P_{GRID,min}=-200\;kW,\;P_{GRID,max}=200\;kW$ denote the maximum GRID interaction capacity.

### 4.3. Objective Function Formulation

The optimization objective is to minimize the total operating cost of the microgrid on a typical day. The total cost includes fuel cost, operating cost, pollutant treatment cost, grid interaction cost, and power imbalance penalty cost. The fitness function is expressed as

(30) $$minF=F_{\mathrm{Fuel}\;}+F_{Op}+F_{Poll}+F_{\mathrm{Grid}\;}+F_{Pen},$$

where F represents the total operating cost; $F_{\mathrm{Fuel}\;}$ denotes the fuel cost; $F_{Op}$ is the operating cost; $F_{Poll}$ represents the pollutant treatment cost; $F_{\mathrm{Grid}\;}$ is the grid interaction cost; $F_{Pen}$ denotes the power imbalance penalty cost.

#### 4.3.1. Fuel Cost $\left(F_{\mathrm{Fuel}\;}\right)$

The fuel cost applies only to the fuel cell (FC), microturbine (MT), and gas supply system (GS) and is calculated based on their output, fuel cost coefficients, and the natural gas price. The expression is given by

(31) $$F_{Fuel}=c\cdot (k_{FC,f}\cdot \sum_{t=1}^{24}P_{FC}(t)+k_{MT,f}\cdot \sum_{t=1}^{24}P_{MT}(t)+k_{GS,f}\cdot \sum_{t=1}^{24}P_{GS}(t)),$$

where $c=2.02\;\$\cdot {kg}^{-1}$ is the natural gas price; $k_{FC,f}=0.2345\;\$\cdot {kW}^{-1},k_{MT,f}=0.4090\;\$\cdot {kW}^{-1},k_{GS,f}=0.6031\;\$\cdot {kW}^{-1}$ are the fuel cost coefficients of FC, MT, and GS, respectively.

#### 4.3.2. Operating Cost $\left(F_{Op}\right)$

The operating cost covers all distributed energy sources in the microgrid and is calculated based on their output and corresponding operating cost coefficients [6]:

(32) FOP=kPV,o∑t=124PPV(t)+kWT,o∑t=124PWT(t)+kFC,o∑t=124PFC(t)+kMT,o∑t=124|PMT(t)|+kGS,o∑t=124PGS(t)+kBT,o∑t=124|PBT(t)|,

where $k_{PV,o}=0.0096\;\$\cdot {kW}^{-1}$, $k_{WT,o}=0.045\;\$\cdot {kW}^{-1},{\;k}_{FC,o}=0.02933\;\$\cdot {kW}^{-1}$, $k_{MT,o}=0.0419\;\$\cdot {kW}^{-1}$, $k_{GS,o}=0.1258\;\$\cdot {kW}^{-1}$, $k_{BT,o}=0.055\;\$\cdot {kW}^{-1}$ are the operating cost coefficients of the respective sources. The absolute values indicate that both MT operation and battery (BT) charging/discharging incur costs.

#### 4.3.3. Pollutant Treatment Cost $\left(F_{Poll}\right)$

The pollutant treatment cost accounts for the emissions of SO_2_, CO_2_, and NO_x_ from FC, MT, GS, and grid power. It is calculated based on emission quantities and treatment fees [6]:

(33) FPoll=∑p=13Cp⋅βFC,p⋅∑t=124PFC(t)+βMT,p⋅∑t=124PMT(t)+∑p=13Cp⋅βGS,p⋅∑t=124PGS(t)+βGRID,p⋅∑t=124PGRID(t),

where p=1,2,3 correspond to SO_2_, CO_2_, and NO_x_, respectively; $C_{1}=6.237\;\$\cdot {kg}^{-1}$, $C_{2}=0.088\;\$\cdot {kg}^{-1}$, $C_{3}=26.46\;\$\cdot {kg}^{-1}$ are the pollutant treatment fees; $\beta_{FC,p},\beta_{MT,p},\beta_{GS,p},\;\beta_{GRID,p}$ are the emission coefficients of each energy source.

#### 4.3.4. Grid Interaction Cost $\left(F_{\mathrm{Grid}\;}\right)$

The grid interaction cost is calculated based on the real-time electricity price, with separate accounting for power purchase and power sale. The expression is as follows [6]:

(34) $$F_{\mathrm{Grid}\;}=\sum_{t\in T_{\mathrm{buy}\;}}P_{\mathrm{GRID}\;}(t)\cdot c_{\mathrm{Grid}\;}(t)+\sum_{t\in T_{\mathrm{sell}\;}}P_{\mathrm{GRID}\;}(t)\cdot c_{\mathrm{Grid}\;}(t),$$

where $T_{\mathrm{buy}\;}=\{t|P_{\mathrm{GRID}\;}\left(t\right)>0\}$ denotes the set of time periods when electricity is purchased, and $T_{\mathrm{sell}\;}=\{t|P_{\mathrm{GRID}\;}(t)<0\}$ represents the set of time periods when electricity is sold. Here, $c_{\mathrm{Grid}\;}(t)$ is the real-time electricity price at time t ($·kW^−1^).

#### 4.3.5. Power Imbalance Penalty Cost $\left(F_{Pen}\right)$

To ensure power balance, a penalty is imposed on the deviation between the total power generation and the load demand. The expression is given by [6]

(35) FPen=∑t=124|PPV(t)+PWT(t)+PFC(t)+PMT(t)+PGS(t)+PBT(t)+PGRID(t)−PLoad (t)|,

where the penalty coefficient is $20\;\$\cdot {kg}^{-1}$, and $P_{\mathrm{Load}\;}(t)$ represents the load demand at time t.

### 4.4. Constraints

At each time period, the sum of the power outputs from all distributed energy sources and the grid interaction power must equal the load demand, which serves as the core constraint [6]:

(36) PLoad t=PPV(t)+PWT(t)+PFC(t)+PMT(t)+PGSt+PBTt+PGRIDt (t=1,2,…,24),

where $P_{\mathrm{Load}\;}\left(t\right)$ is the load demand at time t. In addition, the output of each distributed energy source must remain within its predefined upper and lower bounds, expressed as

(37) $$P_{i,\;\min\;}\leq P_{i}(t)\leq P_{i,\;\max\;}(i=PV,WT,FC,MT,GS,BT,GRID;t=1,2,\ldots ,24),$$

where $P_{i,\;\min\;}$ and $P_{i,\;\max\;}$ denote the lower and upper limits of power output for source i, respectively.

Moreover, the state-of-charge (SOC) of the battery (BT) must be maintained within the safe operating range, following the constraints described in Section 4.2.3.

### 4.5. Results Analysis

**Figure 12:**
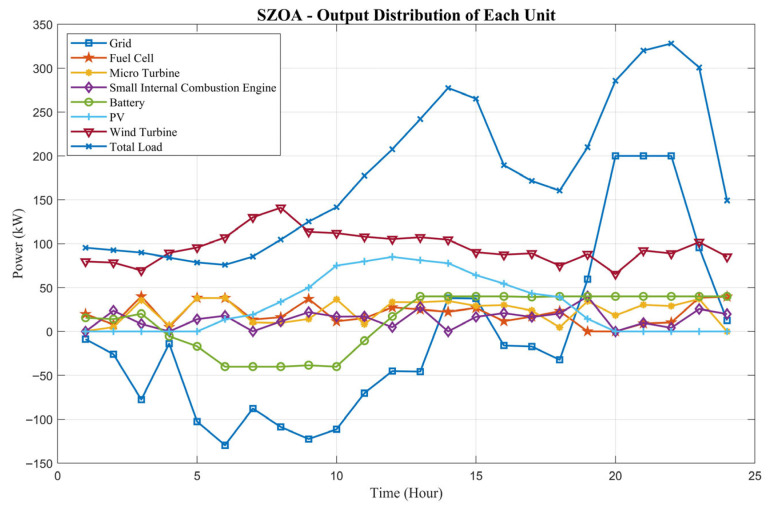
Output power of each power source after SZOA optimization.

**Figure 13:**
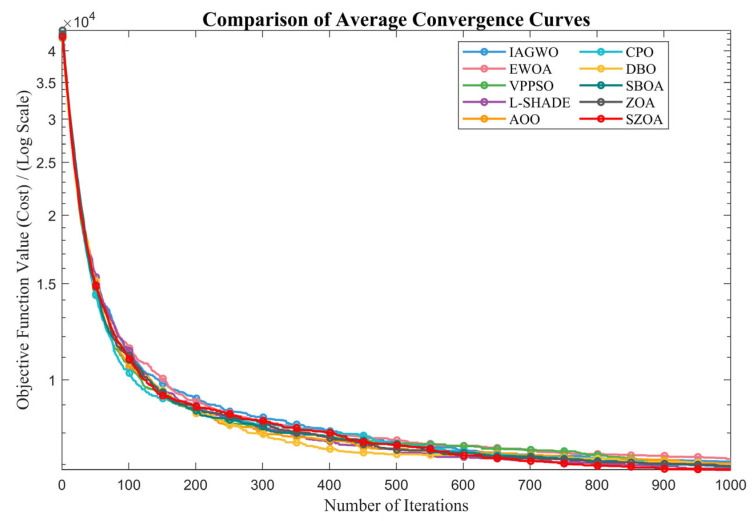
Cost curves obtained by different algorithms.

This study primarily investigates a grid-connected microgrid, with MATLAB employed as the simulation platform. Here, GRID denotes the main power grid. The population size was set to 30, and the number of iterations was fixed at 1000. Other algorithm parameters were configured as described earlier. The interaction power with the main grid was set at 200 kW. A typical day in a certain region of Guangdong Province was selected as the case study. To ensure fairness and reduce randomness, each algorithm was independently executed 30 times. The experimental results were statistically summarized in terms of the maximum value (Max), minimum value (Min), average value (Ave), standard deviation (Std), and ranking (Rank). The best results are highlighted in bold, as shown in Table 8. The output of each distributed energy source is illustrated in Figure 12, and the total operating cost is presented in Figure 13.

Table 8 Using a grid-connected microgrid in Guangdong Province on a typical day as the case study, under unified experimental settings (population size = 30, maximum iterations = 1000, grid interaction power = 200 kW), the scheduling costs of the SZOA were compared with those of nine other algorithms, including IAGWO and EWOA. The statistical indicators include maximum cost (Max), minimum cost (Min), mean cost (Mean), standard deviation (Std), and ranking (Rank). The results show that the SZOA consistently outperforms all competitors. Its minimum cost is USD 5165.96, significantly lower than that for L-SHADE (USD 5717.85) and IAGWO (USD 6260.32), and clearly superior to the that of the original ZOA (USD 6171.33). The average cost reaches USD 6853.07, which is lower than that for L-SHADE (USD 6880.38) and EWOA (USD 7166.66). Moreover, its standard deviation is only USD 448.53, the lowest among all algorithms, indicating that the SZOA can repeatedly deliver high-quality scheduling solutions across multiple independent runs. It is worth noting that while the SZOA excels in mean and standard deviation, it does not achieve the lowest maximum cost (USD 8291.60), and is even slightly higher than the results for algorithms like AOO (USD 8137.78) in a few runs. This is attributed to the SZOA’s design focus on balancing global exploration and local exploitation: its multi-population cooperative search mechanism integrates differential vectors from individuals with different fitness levels (global best, suboptimal, worst, random individuals) to avoid premature convergence, and the vertical crossover–mutation strategy introduces random perturbations to enhance high-dimensional optimization capability; these designs, while ensuring overall stability and low average cost, may occasionally lead to temporary deviations toward suboptimal regions in individual runs. In contrast, algorithms like the AOO may adopt a more conservative local exploitation strategy that narrows the search scope to reduce extreme maximum values, but this often comes at the cost of sacrificing global optimization potential, as reflected in their higher mean costs and standard deviations. Nevertheless, the SZOA’s significantly lower mean and standard deviation confirm that such extreme cases are rare and do not weaken its overall superiority. Overall, the SZOA ranks first, far ahead of the original ZOA (ranked sixth) and DBO (ranked tenth), demonstrating its superior cost-control capability and solution stability for practical microgrid scheduling problems.

With a 24 h timeline, Figure 12 intuitively illustrates the SZOA’s coordinated scheduling strategy across multiple distributed energy sources, including photovoltaic (PV) generation, wind turbine (WT), fuel cell (FC), and battery storage (BT). The curves show that the SZOA dynamically adjusts the operation of each source based on renewable generation profiles. From 8:00 to 18:00, PV output increases with solar irradiance, peaking near 35 kW, while WT output remains around 30–40 kW. During this period, the SZOA reduces the output of controllable sources such as FC (maintained at 5–10 kW) and microturbine (MT, close to 0 kW), while charging BT at 10–20 kW to store surplus renewable energy, thereby reducing reliance on costly sources. From 19:00 to 7:00, PV output drops to 0 kW, and WT output falls to 5–10 kW. The SZOA then discharges BT at −20 to −30 kW (negative sign indicating discharge) to cover the load gap, while increasing FC output to 15–20 kW to minimize grid purchase. This scheduling strategy maximizes the use of zero-fuel-cost renewables and ensures supply–demand balance through the complementary operation of storage and controllable units, directly supporting the cost advantage reflected in Table 8.

Figure 13 further validates the SZOA’s superiority from a dynamic perspective. The horizontal axis represents the number of iterations (0–1000), and the vertical axis indicates the scheduling cost. The results show that the SZOA leads in both convergence speed and final cost. In the early stage (0–200 iterations), the SZOA rapidly reduces the cost from 3.5 × 10^4^ to 3.1 × 10^4^, while the results for the original ZOA and CPO remain at 3.3 × 10^4^ to 3.4 × 10^4^. During the mid-stage (200–600 iterations), the SZOA continues its steady decline, gradually widening the performance gap between other algorithms. In the late stage (600–1000 iterations), the SZOA converges first, stabilizing around 2.9 × 10^4^, whereas the original ZOA and DBO converge slowly to values that are 10–15% higher. This advantage stems from the SZOA’s multi-population cooperative search mechanism, which efficiently explores the global optimum and avoids premature convergence caused by single-directional search, while its vertical crossover–mutation strategy enhances the accuracy of high-dimensional scheduling across 24 h. Together, these mechanisms enable breakthroughs in both cost reduction and convergence efficiency.

The quantitative evidence in Table 8, along with the visual insights from Figure 12 and Figure 13, jointly demonstrate that the SZOA not only excels in precision and stability for numerical optimization tasks but also effectively adapts to practical microgrid scheduling. By enabling multi-source coordinated scheduling to reduce operational costs and achieving rapid convergence to improve application efficiency, the SZOA provides a reliable optimization solution to ensure the economic and stable operation of grid-connected microgrids.

## 5. Summary and Prospect

This study focuses on the core demand of “economic–low-carbon” synergistic optimization scheduling in microgrids. To address the shortcomings of the traditional Zebra Optimization Algorithm (ZOA)—namely the reliance on a single search mechanism, insufficient inter-dimensional coordination, and coarse boundary handling when solving complex high-dimensional problems—we propose a Synergistic Zebra Optimization Algorithm (SZOA). Its effectiveness is verified through numerical experiments and engineering applications. The main conclusions are as follows:

From the perspective of algorithmic improvements, the SZOA achieves performance breakthroughs through three core strategies. First, the multi-population synergistic search mechanism integrates difference vectors from the global best, suboptimal, worst, and random individuals. By dynamically assigning learning factors based on Euclidean distance, it builds a comprehensive search direction and effectively mitigates the rapid loss of population diversity caused by the reliance on a single “leader zebra” in the standard ZOA, thus providing multi-source support for global exploration. Second, the vertical crossover–mutation strategy reorganizes dimensions within individuals and introduces random perturbations, breaking the limitation of “dimension-independent updates” in traditional algorithms. This enhances complementarity and coordination across dimensions in high-dimensional problems, preventing certain dimensions from falling into local optima. Third, the leader-guided boundary control strategy transforms fixed boundaries into dynamic constraint intervals centered on the population’s best individual. This replaces the “direct truncation” method, ensuring continuity and rationality in position updates, while reducing local optima traps near the boundary.

For numerical validation, the SZOA was tested on the CEC2017 benchmark set (dimension d = 30) and the CEC2022 benchmark set (dimensions d = 10 and d = 20) and compared against nine algorithms, including the Improved Adaptive Grey Wolf Optimizer (IAGWO), the Enhanced Whale Optimization Algorithm (EWOA), and the original ZOA. Results show that the SZOA consistently achieved the best performance across unimodal, multimodal, hybrid, and composite functions. For example, in the CEC2017 F1 (unimodal) test, the average objective value obtained by the SZOA was 5.2887 × 10^3^, far lower than the original ZOA’s 1.0170 × 10^10^. In the F30 (multimodal) test, the SZOA achieved an average value of just 5.8597 × 10^3^, only one-sixteenth of the value of the original ZOA, with a significantly lower standard deviation. Wilcoxon rank-sum and Friedman mean-rank tests further confirmed that the improvements in the SZOA are statistically significant. The SZOA consistently maintained a leading position across dimensions, with the lowest average ranks (1.20 for CEC2017 and 1.25 for CEC2022 with d = 20).

In terms of engineering applications, the SZOA was applied to the grid-connected microgrid scheduling problem. The microgrid model incorporates photovoltaic generation (PV), wind power (WT), fuel cells (FC), microturbines (MT), battery storage (BT), and grid interaction. A dual-objective framework was established with “economic cost minimization” and “low-carbon emission reduction”. The objective functions included fuel cost, operation cost, and pollution treatment cost, subject to multiple constraints. Simulation results based on a typical day in Guangdong Province show that the SZOA-optimized microgrid achieved a minimum operating cost of USD 5165.96, an average cost of USD 6853.07, and a standard deviation of only USD 448.53—all superior compared to the results for the other algorithms. Furthermore, the SZOA effectively coordinated the outputs of the distributed energy sources. During the daytime, it maximized PV utilization (peaking near 35 kW) and WT output (30–40 kW), while reducing reliance on fossil fuel generators such as FC and MT. At night, BT discharge (–20 to –30 kW) compensated for load gaps, thereby reducing fossil fuel consumption and pollutant emissions and realizing the “economic–low-carbon” synergy in microgrid optimization.

Although this study provides a reliable solution through algorithmic innovations and engineering validation of the SZOA, several promising extensions remain. This study still exhibits certain limitations. On the one hand, the SZOA-based microgrid scheduling model adopts deterministic typical daily data of renewable energy output and load demand, lacking an optimization mechanism to cope with uncertainties like extreme weather or sudden load changes, which may reduce the solution’s robustness. On the other hand, the SZOA is only applied to single grid-connected microgrid scheduling, and its adaptability to complex multi-microgrid interconnected systems with power trading and shared energy storage remains untested. Additionally, the slight increase in computational overhead due to its three enhancement strategies may make it difficult to meet real-time scheduling needs in large-scale, high-dimensional microgrid scenarios.

Future research may incorporate interval optimization and robust optimization methods, building uncertainty-aware scheduling models that consider extreme weather and sudden load fluctuations to enhance robustness. The SZOA could also be extended to interconnected multi-microgrid systems to optimize power trading and storage allocation, improving regional energy system performance. Moreover, based on multi-objective optimization theory, the objective system could be expanded to include equipment lifetime loss and supply reliability, with tailored multi-objective solving strategies. Finally, engineering modules of the SZOA could be developed, integrated with edge computing and real-time data acquisition technologies, to promote its transition into practical engineering applications.

## Figures and Tables

**Table 1:** Comparison of algorithm parameter settings.

Algorithms	Name of the Parameter	Value of the Parameter
IAGWO	$v_{rand}\left(t\right),a,\;\omega ,\theta$	[−20, 20], [0, 2], [0.3, 0.9], 0.5
EWOA	a	[0, 2]
VPPSO	$c_{1},c_{2},\;w,\alpha ,N_{1},N_{2}$	2, 2, 0.8, [1, 0], 0.15, 0.15
L-SHADE	${NP}_{init},{NP}_{min},P,\left|A\right|,H$	18, 4, 0.11, 2.6, 6
AOO	K,θ	[0.5, 1.5],[0,π]
CPO	$\alpha ,N_{min},T_{f},T$	0.1, 80, 0.5, 2
DBO	$P_{percent}$	0.2
SBOA	$CF,K,R_{1},R_{2}$	$\left[0\;,1\right],\left\{1,\;2\right\},[0,\;1],[0,\;1]$
ZOA	${I,P}_{S},R$	$\left\{1,\;2\right\},\left[0,\;1\right],0.1$
SZOA	${I,P}_{S},F,\alpha ,\beta$	$\left\{1,\;2\right\},\left[0,\;1\right],\left[0,\;0.5\right],\left[0,\;1\right],[-1,\;1]$

**Table 2:** Experimental results of CEC2017 (d = 30).

Function	Metric	IAGWO	EWOA	VPPSO	L-SHADE	AOO	CPO	DBO	SBOA	ZOA	SZOA
F1	Ave	1.5244 × 10^6^	4.6039 × 10^9^	4.5248 × 10^6^	6.3710 × 10^4^	1.0494 × 10^5^	5.3611 × 10^5^	2.5127 × 10^8^	1.8914 × 10^4^	1.2199 × 10^10^	**4.1226 × 10^3^**
	Std	4.5746 × 10^5^	3.5959 × 10^9^	1.7663 × 10^7^	7.2267 × 10^4^	4.9323 × 10^4^	3.8778 × 10^5^	1.4611 × 10^8^	1.9996 × 10^4^	4.7943 × 10^9^	**4.8126 × 10^3^**
F2	Ave	1.3668 × 10^38^	5.4413 × 10^35^	5.2352 × 10^25^	1.3028 × 10^18^	2.9077 × 10^16^	1.4465 × 10^21^	2.2612 × 10^32^	2.5240 × 10^17^	1.8366 × 10^43^	**2.2886 × 10^10^**
	Std	7.4823 × 10^38^	2.6460 × 10^36^	2.2358 × 10^26^	4.3033 × 10^18^	6.5305 × 10^16^	7.3542 × 10^21^	8.3892 × 10^32^	6.3293 × 10^17^	1.0059 × 10^44^	**6.3635 × 10^10^**
F3	Ave	6.2781 × 10^4^	1.9292 × 10^5^	4.6825 × 10^4^	4.4769 × 10^4^	2.5402 × 10^4^	6.2797 × 10^4^	9.4449 × 10^4^	2.5463 × 10^4^	5.4681 × 10^4^	**6.0137 × 10^2^**
	Std	1.7992 × 10^4^	3.1529 × 10^4^	1.2695 × 10^4^	4.5121 × 10^4^	8.6872 × 10^3^	1.2894 × 10^4^	2.8209 × 10^4^	8.7146 × 10^3^	8.3742 × 10^3^	**2.8726 × 10^2^**
F4	Ave	6.0624 × 10^2^	1.0774 × 10^3^	5.2510 × 10^2^	4.9847 × 10^2^	5.0005 × 10^2^	5.1673 × 10^2^	7.0080 × 10^2^	5.1105 × 10^2^	2.0751 × 10^3^	**4.6669 × 10^2^**
	Std	1.1908 × 10^2^	3.9998 × 10^2^	2.4294 × 10^1^	3.0070 × 10^1^	**1.8273 × 10^1^**	2.0131 × 10^1^	1.9463 × 10^2^	2.2654 × 10^1^	1.1372 × 10^3^	3.5660 × 10^1^
F5	Ave	6.5579 × 10^2^	7.5763 × 10^2^	6.4385 × 10^2^	**5.6882 × 10^2^**	6.2320 × 10^2^	6.9432 × 10^2^	7.5995 × 10^2^	5.8086 × 10^2^	7.1319 × 10^2^	5.9957 × 10^2^
	Std	3.2033 × 10^1^	3.9886 × 10^1^	3.0963 × 10^1^	**1.1587 × 10^1^**	3.0881 × 10^1^	1.9323 × 10^1^	6.0325 × 10^1^	2.1650 × 10^1^	3.0634 × 10^1^	1.9356 × 10^1^
F6	Ave	6.2265 × 10^2^	6.5547 × 10^2^	6.3861 × 10^2^	6.0173 × 10^2^	6.3029 × 10^2^	6.0193 × 10^2^	6.5040 × 10^2^	6.0239 × 10^2^	6.5548 × 10^2^	**6.0012 × 10^2^**
	Std	1.0170 × 10^1^	1.4033 × 10^1^	7.0614 × 10^00^	1.5633 × 10^00^	9.7225 × 10^00^	1.1057 × 10^00^	1.0003 × 10^1^	2.2134 × 10^00^	6.5850 × 10^00^	**1.0181 × 10^−1^**
F7	Ave	9.2371 × 10^2^	1.1706 × 10^3^	9.3198 × 10^2^	8.4327 × 10^2^	8.7374 × 10^2^	9.4959 × 10^2^	1.0362 × 10^3^	8.5751 × 10^2^	1.1433 × 10^3^	**8.3959 × 10^2^**
	Std	4.3907 × 10^1^	1.0925 × 10^2^	5.2446 × 10^1^	2.8340 × 10^1^	4.1798 × 10^1^	**1.9759 × 10^1^**	8.6477 × 10^1^	5.2460 × 10^1^	6.8659 × 10^1^	2.0040 × 10^1^
F8	Ave	9.2187 × 10^2^	1.0421 × 10^3^	9.2018 × 10^2^	**8.6768 × 10^2^**	9.1332 × 10^2^	9.7702 × 10^2^	1.0278 × 10^3^	8.7417 × 10^2^	9.6629 × 10^2^	8.9228 × 10^2^
	Std	2.8122 × 10^1^	5.1893 × 10^1^	2.7378 × 10^1^	1.7412 × 10^1^	3.0967 × 10^1^	**1.4078 × 10^1^**	4.1740 × 10^1^	1.6994 × 10^1^	2.5352 × 10^1^	1.7588 × 10^1^
F9	Ave	4.3174 × 10^3^	1.1535 × 10^4^	3.8537 × 10^3^	1.4218 × 10^3^	3.4267 × 10^3^	1.2221 × 10^3^	6.6208 × 10^3^	1.6648 × 10^3^	4.7744 × 10^3^	**1.1681 × 10^3^**
	Std	1.0584 × 10^3^	5.4983 × 10^3^	9.6479 × 10^2^	3.8341 × 10^2^	1.2216 × 10^3^	**2.4829 × 10^2^**	1.9500 × 10^3^	6.6687 × 10^2^	8.8729 × 10^2^	4.6167 × 10^2^
F10	Ave	4.3584 × 10^3^	8.0823 × 10^3^	5.0082 × 10^3^	4.5776 × 10^3^	4.8212 × 10^3^	7.6378 × 10^3^	6.5128 × 10^3^	4.3798 × 10^3^	5.3130 × 10^3^	**4.1657 × 10^3^**
	Std	7.5915 × 10^2^	1.0174 × 10^3^	7.7631 × 10^2^	3.9071 × 10^2^	8.1889 × 10^2^	**3.4686 × 10^2^**	1.2114 × 10^3^	6.0400 × 10^2^	6.5244 × 10^2^	3.6102 × 10^2^
F11	Ave	1.9217 × 10^3^	4.1304 × 10^3^	1.4614 × 10^3^	1.2578 × 10^3^	1.2880 × 10^3^	1.2768 × 10^3^	2.0207 × 10^3^	1.2136 × 10^3^	2.4270 × 10^3^	**1.1587 × 10^3^**
	Std	9.1459 × 10^2^	2.0765 × 10^3^	1.2439 × 10^2^	6.3990 × 10^1^	4.3955 × 10^1^	**1.9643 × 10^1^**	8.3337 × 10^2^	4.4503 × 10^1^	8.7833 × 10^2^	2.8434 × 10^1^
F12	Ave	3.7109 × 10^7^	1.7730 × 10^8^	3.3573 × 10^7^	2.4287 × 10^5^	1.2234 × 10^7^	1.1932 × 10^6^	9.8569 × 10^7^	1.3046 × 10^6^	8.5554 × 10^8^	**6.8360 × 10^4^**
	Std	6.7532 × 10^7^	4.2921 × 10^8^	2.6992 × 10^7^	2.9906 × 10^5^	1.1638 × 10^7^	6.0858 × 10^5^	1.9600 × 10^8^	1.4049 × 10^6^	9.5446 × 10^8^	**8.8217 × 10^4^**
F13	Ave	3.6350 × 10^7^	7.2570 × 10^7^	1.1043 × 10^5^	1.7864 × 10^4^	9.0933 × 10^4^	2.3323 × 10^4^	5.4961 × 10^6^	2.1156 × 10^4^	2.2673 × 10^8^	**1.8459 × 10^3^**
	Std	1.6464 × 10^8^	3.7989 × 10^8^	6.8151 × 10^4^	1.2712 × 10^4^	6.4594 × 10^4^	1.9768 × 10^4^	1.2079 × 10^7^	1.9802 × 10^4^	3.1756 × 10^8^	**9.4920 × 10^2^**
F14	Ave	6.3359 × 10^5^	6.1416 × 10^5^	1.2850 × 10^5^	1.9735 × 10^3^	6.1639 × 10^4^	2.1292 × 10^3^	3.5638 × 10^5^	3.8961 × 10^4^	4.1496 × 10^5^	**1.4696 × 10^3^**
	Std	5.9986 × 10^5^	4.9960 × 10^5^	9.5971 × 10^4^	1.9044 × 10^3^	5.4624 × 10^4^	7.3541 × 10^2^	4.5703 × 10^5^	3.5334 × 10^4^	3.7730 × 10^5^	**9.2980 × 10^00^**
F15	Ave	4.3619 × 10^3^	4.6265 × 10^4^	4.8870 × 10^4^	2.0294 × 10^3^	5.4074 × 10^4^	4.5251 × 10^3^	7.2827 × 10^4^	1.5602 × 10^4^	3.0615 × 10^6^	**1.5636 × 10^3^**
	Std	2.9091 × 10^3^	3.0890 × 10^4^	3.8264 × 10^4^	1.7160 × 10^2^	4.7448 × 10^4^	2.4422 × 10^3^	6.5447 × 10^4^	1.5495 × 10^4^	4.4587 × 10^6^	**2.5597 × 10^1^**
F16	Ave	3.1012 × 10^3^	3.4541 × 10^3^	2.9472 × 10^3^	2.5684 × 10^3^	2.6672 × 10^3^	3.1331 × 10^3^	3.5022 × 10^3^	2.4367 × 10^3^	3.2951 × 10^3^	**2.3610 × 10^3^**
	Std	3.1072 × 10^2^	3.9189 × 10^2^	3.5208 × 10^2^	2.2255 × 10^2^	3.6478 × 10^2^	1.7740 × 10^2^	4.2595 × 10^2^	2.5266 × 10^2^	4.4395 × 10^2^	**1.7147 × 10^2^**
F17	Ave	2.3745 × 10^3^	2.6624 × 10^3^	2.2095 × 10^3^	1.9726 × 10^3^	2.1191 × 10^3^	2.0559 × 10^3^	2.6912 × 10^3^	1.9728 × 10^3^	2.4354 × 10^3^	**1.9181 × 10^3^**
	Std	2.2999 × 10^2^	2.3621 × 10^2^	1.8711 × 10^2^	1.5046 × 10^2^	1.6027 × 10^2^	1.1615 × 10^2^	2.2771 × 10^2^	1.7062 × 10^2^	3.0927 × 10^2^	**9.8761 × 10^1^**
F18	Ave	1.1873 × 10^6^	6.9104 × 10^6^	1.1822 × 10^6^	6.0013 × 10^4^	9.3563 × 10^5^	1.2564 × 10^5^	4.0195 × 10^6^	5.5359 × 10^5^	4.3195 × 10^6^	**1.8579 × 10^3^**
	Std	1.5335 × 10^6^	8.7040 × 10^6^	1.1439 × 10^6^	6.2147 × 10^4^	1.7007 × 10^6^	6.2903 × 10^4^	5.0887 × 10^6^	5.3355 × 10^5^	7.2862 × 10^6^	**9.6309 × 10^00^**
F19	Ave	1.1384 × 10^4^	1.0095 × 10^6^	1.7902 × 10^6^	3.2277 × 10^3^	5.8442 × 10^5^	5.7917 × 10^3^	3.7402 × 10^6^	1.4104 × 10^4^	2.9028 × 10^6^	**1.9354 × 10^3^**
	Std	1.0027 × 10^4^	3.0252 × 10^6^	1.1465 × 10^6^	3.4826 × 10^3^	5.7764 × 10^5^	4.3034 × 10^3^	5.4768 × 10^6^	1.7529 × 10^4^	2.3814 × 10^6^	**6.9391 × 10^00^**
F20	Ave	2.6024 × 10^3^	2.7232 × 10^3^	2.4864 × 10^3^	2.4213 × 10^3^	2.4727 × 10^3^	2.4894 × 10^3^	2.7446 × 10^3^	2.2936 × 10^3^	2.4586 × 10^3^	**2.2526 × 10^3^**
	Std	1.6334 × 10^2^	1.6168 × 10^2^	1.3680 × 10^2^	1.2513 × 10^2^	1.8715 × 10^2^	1.3046 × 10^2^	2.4139 × 10^2^	1.7624 × 10^2^	1.1224 × 10^2^	**7.9703 × 10^1^**
F21	Ave	2.4621 × 10^3^	2.5458 × 10^3^	2.4341 × 10^3^	2.3737 × 10^3^	2.4184 × 10^3^	2.4842 × 10^3^	2.5589 × 10^3^	**2.3601 × 10^3^**	2.4988 × 10^3^	2.3909 × 10^3^
	Std	3.8402 × 10^1^	5.2900 × 10^1^	4.1836 × 10^1^	1.4410 × 10^1^	2.7252 × 10^1^	**1.2954 × 10^1^**	5.0181 × 10^1^	1.6627 × 10^1^	3.4217 × 10^1^	1.5076 × 10^1^
F22	Ave	4.2267 × 10^3^	7.1957 × 10^3^	3.2361 × 10^3^	2.9068 × 10^3^	4.2253 × 10^3^	**2.3093 × 10^3^**	4.9559 × 10^3^	2.7634 × 10^3^	5.6930 × 10^3^	2.4286 × 10^3^
	Std	2.1182 × 10^3^	2.8054 × 10^3^	1.8269 × 10^3^	1.2668 × 10^3^	1.9855 × 10^3^	**3.5699 × 10^00^**	2.6782 × 10^3^	1.2131 × 10^3^	1.0843 × 10^3^	7.0072 × 10^2^
F23	Ave	3.1280 × 10^3^	2.9167 × 10^3^	2.8133 × 10^3^	**2.7198 × 10^3^**	2.7983 × 10^3^	2.8477 × 10^3^	3.0262 × 10^3^	2.7341 × 10^3^	3.2618 × 10^3^	2.7443 × 10^3^
	Std	1.6160 × 10^2^	7.0933 × 10^1^	6.6073 × 10^1^	**1.4312 × 10^1^**	3.9819 × 10^1^	1.7908 × 10^1^	7.0577 × 10^1^	2.7589 × 10^1^	8.4146 × 10^1^	1.6875 × 10^1^
F24	Ave	3.3015 × 10^3^	3.0958 × 10^3^	2.9531 × 10^3^	**2.8885 × 10^3^**	2.9601 × 10^3^	3.0237 × 10^3^	3.2200 × 10^3^	2.8913 × 10^3^	3.4748 × 10^3^	2.9296 × 10^3^
	Std	1.2400 × 10^2^	5.4159 × 10^1^	5.3204 × 10^1^	**1.5487 × 10^1^**	4.7355 × 10^1^	1.9107 × 10^1^	1.0131 × 10^2^	1.9315 × 10^1^	1.0043 × 10^2^	2.1624 × 10^1^
F25	Ave	2.9510 × 10^3^	3.1264 × 10^3^	2.9475 × 10^3^	**2.8917 × 10^3^**	2.9182 × 10^3^	2.9161 × 10^3^	2.9918 × 10^3^	2.9085 × 10^3^	3.2238 × 10^3^	2.8932 × 10^3^
	Std	2.8943 × 10^1^	1.1572 × 10^2^	2.6809 × 10^1^	**6.5078 × 10^00^**	2.4562 × 10^1^	2.0995 × 10^1^	8.2882 × 10^1^	2.0536 × 10^1^	1.5077 × 10^2^	1.5482 × 10^1^
F26	Ave	5.2430 × 10^3^	6.6261 × 10^3^	4.7339 × 10^3^	4.3952 × 10^3^	4.6515 × 10^3^	5.2042 × 10^3^	6.9401 × 10^3^	4.1294 × 10^3^	8.2226 × 10^3^	**3.4673 × 10^3^**
	Std	1.7789 × 10^3^	8.1709 × 10^2^	1.3499 × 10^3^	**3.3330 × 10^2^**	1.1741 × 10^3^	9.8576 × 10^2^	5.9495 × 10^2^	6.9928 × 10^2^	5.7639 × 10^2^	8.8892 × 10^2^
F27	Ave	3.2409 × 10^3^	3.2770 × 10^3^	3.3096 × 10^3^	3.2255 × 10^3^	3.2585 × 10^3^	3.2811 × 10^3^	3.3262 × 10^3^	3.2242 × 10^3^	3.9264 × 10^3^	**3.2161 × 10^3^**
	Std	1.1665 × 10^2^	2.9582 × 10^1^	6.5419 × 10^1^	1.1311 × 10^1^	2.5713 × 10^1^	**1.0874 × 10^1^**	7.6799 × 10^1^	1.5773 × 10^1^	2.4374 × 10^2^	1.6853 × 10^1^
F28	Ave	3.3895 × 10^3^	3.7913 × 10^3^	3.3101 × 10^3^	3.2431 × 10^3^	3.2455 × 10^3^	3.2717 × 10^3^	3.6709 × 10^3^	3.2562 × 10^3^	3.9855 × 10^3^	**3.1902 × 10^3^**
	Std	1.5764 × 10^2^	5.6498 × 10^2^	3.2125 × 10^1^	2.7223 × 10^1^	**2.3319 × 10^1^**	2.8693 × 10^1^	7.2465 × 10^2^	4.0807 × 10^1^	4.3154 × 10^2^	5.0155 × 10^1^
F29	Ave	3.9866 × 10^3^	4.5068 × 10^3^	4.2945 × 10^3^	3.6744 × 10^3^	4.0610 × 10^3^	4.0168 × 10^3^	4.3787 × 10^3^	3.6288 × 10^3^	5.2046 × 10^3^	**3.5444 × 10^3^**
	Std	2.9297 × 10^2^	3.7783 × 10^2^	2.6930 × 10^2^	1.2784 × 10^2^	2.7728 × 10^2^	1.9084 × 10^2^	4.1399 × 10^2^	1.8601 × 10^2^	6.2516 × 10^2^	**8.6915 × 10^1^**
F30	Ave	3.5606 × 10^4^	6.7377 × 10^6^	8.7773 × 10^6^	1.6968 × 10^4^	3.8524 × 10^6^	1.1992 × 10^5^	5.0171 × 10^6^	3.7646 × 10^4^	3.2705 × 10^7^	**6.3109 × 10^3^**
	Std	1.5936 × 10^5^	2.0562 × 10^7^	5.5082 × 10^6^	1.0546 × 10^4^	1.9331 × 10^6^	5.8851 × 10^4^	7.0350 × 10^6^	7.4693 × 10^4^	3.0315 × 10^7^	**2.1170 × 10^3^**

**Table 3:** Experimental results of CEC2022 (d = 10).

Function	Metric	IAGWO	EWOA	VPPSO	L-SHADE	AOO	CPO	DBO	SBOA	ZOA	SZOA
F1	Ave	3.3725 × 10^2^	9.4698 × 10^3^	3.1142 × 10^2^	3.0000 × 10^2^	3.0000 × 10^2^	4.0417 × 10^2^	2.2860 × 10^3^	3.0005 × 10^2^	1.3639 × 10^3^	**3.0000 × 10^2^**
	Std	4.4184 × 10^1^	4.4609 × 10^3^	3.3112 × 10^1^	8.8000 × 10^−6^	4.0931 × 10^−3^	9.7996 × 10^1^	2.4399 × 10^3^	9.0636 × 10^−2^	1.6541 × 10^3^	**2.5856 × 10^−14^**
F2	Ave	4.4006 × 10^2^	4.4276 × 10^2^	4.0633 × 10^2^	4.0620 × 10^2^	4.1654 × 10^2^	**4.0092 × 10^2^**	4.4790 × 10^2^	4.1122 × 10^2^	4.4871 × 10^2^	4.0099 × 10^2^
	Std	3.4375 × 10^1^	3.8463 × 10^1^	5.3605 × 10^00^	2.9321 × 10^00^	2.6710 × 10^1^	**2.0484 × 10^00^**	5.2131 × 10^1^	2.0578 × 10^1^	3.1618 × 10^1^	2.4711 × 10^00^
F3	Ave	6.0035 × 10^2^	6.1018 × 10^2^	6.0872 × 10^2^	6.0000 × 10^2^	6.0243 × 10^2^	6.0000 × 10^2^	6.1219 × 10^2^	6.0000 × 10^2^	6.2131 × 10^2^	**6.0000 × 10^2^**
	Std	1.9909 × 10^−1^	6.3092 × 10^00^	5.9292 × 10^00^	1.9907 × 10^−3^	1.6905 × 10^00^	2.3815 × 10^−3^	9.7161 × 10^00^	1.4554 × 10^−3^	6.9659 × 10^00^	**5.0622 × 10^−4^**
F4	Ave	8.2246 × 10^2^	8.3488 × 10^2^	8.1729 × 10^2^	8.0950 × 10^2^	8.1960 × 10^2^	8.2059 × 10^2^	8.3693 × 10^2^	8.1181 × 10^2^	8.1508 × 10^2^	**8.0769 × 10^2^**
	Std	1.1397 × 10^1^	1.4262 × 10^1^	6.2956 × 10^00^	4.5058 × 10^00^	8.2027 × 10^00^	5.6574 × 10^00^	1.1860 × 10^1^	4.0540 × 10^00^	4.8699 × 10^00^	**3.2353 × 10^00^**
F5	Ave	9.2617 × 10^2^	1.0653 × 10^3^	9.1204 × 10^2^	9.0047 × 10^2^	9.0089 × 10^2^	**9.0000 × 10^2^**	9.8544 × 10^2^	9.0004 × 10^2^	1.0794 × 10^3^	9.0002 × 10^2^
	Std	4.4024 × 10^1^	1.5426 × 10^2^	9.0764 × 10^00^	7.1948 × 10^−1^	2.7566 × 10^00^	**6.1855 × 10^−4^**	1.1008 × 10^2^	1.2982 × 10^−1^	8.8516 × 10^1^	1.0075 × 10^−1^
F6	Ave	8.2218 × 10^4^	5.3832 × 10^3^	3.7893 × 10^3^	1.8248 × 10^3^	4.2380 × 10^3^	1.8240 × 10^3^	5.3424 × 10^3^	4.2883 × 10^3^	3.5250 × 10^3^	**1.8014 × 10^3^**
	Std	4.3538 × 10^5^	1.9917 × 10^3^	2.2038 × 10^3^	2.7038 × 10^1^	2.3126 × 10^3^	1.2691 × 10^1^	2.4455 × 10^3^	2.2226 × 10^3^	1.7980 × 10^3^	**4.5770 × 10^−1^**
F7	Ave	2.0149 × 10^3^	2.0305 × 10^3^	2.0402 × 10^3^	2.0078 × 10^3^	2.0311 × 10^3^	2.0115 × 10^3^	2.0423 × 10^3^	2.0152 × 10^3^	2.0507 × 10^3^	**2.0064 × 10^3^**
	Std	9.1716 × 10^00^	8.6414 × 10^00^	1.3231 × 10^1^	7.2525 × 10^00^	8.8938 × 10^00^	4.8551 × 10^00^	2.1366 × 10^1^	9.5310 × 10^00^	2.0151 × 10^1^	**4.5550 × 10^00^**
F8	Ave	2.2208 × 10^3^	2.2267 × 10^3^	2.2254 × 10^3^	2.2181 × 10^3^	2.2247 × 10^3^	2.2189 × 10^3^	2.2325 × 10^3^	2.2184 × 10^3^	2.2534 × 10^3^	**2.2160 × 10^3^**
	Std	**3.2012 × 10^00^**	4.2930 × 10^00^	6.3602 × 10^00^	6.5849 × 10^00^	4.7967 × 10^00^	4.8104 × 10^00^	2.3479 × 10^1^	7.5728 × 10^00^	5.7295 × 10^1^	7.5852 × 10^00^
F9	Ave	**2.5236 × 10^3^**	2.5368 × 10^3^	2.5461 × 10^3^	2.5293 × 10^3^	2.5343 × 10^3^	2.5293 × 10^3^	2.5620 × 10^3^	2.5293 × 10^3^	2.6236 × 10^3^	2.5293 × 10^3^
	Std	3.0975 × 10^1^	2.5832 × 10^1^	3.7978 × 10^1^	3.9608 × 10^−13^	2.6810 × 10^1^	4.2243 × 10^−3^	4.7760 × 10^1^	**8.4444 × 10^−14^**	4.7104 × 10^1^	1.1942 × 10^−13^
F10	Ave	2.5571 × 10^3^	**2.5012 × 10^3^**	2.5429 × 10^3^	2.5441 × 10^3^	2.5838 × 10^3^	2.5235 × 10^3^	2.5282 × 10^3^	2.5406 × 10^3^	2.5676 × 10^3^	2.5361 × 10^3^
	Std	6.1652 × 10^1^	**3.3758 × 10^−1^**	5.6686 × 10^1^	6.0400 × 10^1^	1.4100 × 10^2^	4.6836 × 10^1^	5.4972 × 10^1^	5.3767 × 10^1^	6.4607 × 10^1^	5.1528 × 10^1^
F11	Ave	2.7466 × 10^3^	2.7340 × 10^3^	2.6929 × 10^3^	2.6485 × 10^3^	2.7062 × 10^3^	**2.6000 × 10^3^**	2.7621 × 10^3^	2.7168 × 10^3^	2.8581 × 10^3^	2.6550 × 10^3^
	Std	1.4308 × 10^2^	8.3369 × 10^1^	1.4927 × 10^2^	9.2558 × 10^1^	1.7248 × 10^2^	**1.3159 × 10^−2^**	1.4998 × 10^2^	1.3670 × 10^2^	2.7139 × 10^2^	1.1475 × 10^2^
F12	Ave	2.8804 × 10^3^	2.8677 × 10^3^	2.8639 × 10^3^	2.8637 × 10^3^	2.8646 × 10^3^	2.8655 × 10^3^	2.8708 × 10^3^	**2.8631 × 10^3^**	2.9697 × 10^3^	2.8642 × 10^3^
	Std	2.4943 × 10^1^	2.7478 × 10^00^	1.2553 × 10^00^	1.3349 × 10^00^	1.4849 × 10^00^	**1.1286 × 10^00^**	1.4039 × 10^1^	1.2044 × 10^00^	4.5658 × 10^1^	1.1538 × 10^00^

**Table 4:** Experimental results of CEC2022 (d = 20).

Function	Metric	IAGWO	EWOA	VPPSO	L-SHADE	AOO	CPO	DBO	SBOA	ZOA	SZOA
F1	Ave	5.1741 × 10^4^	6.4105 × 10^3^	7.0651 × 10^3^	4.3381 × 10^2^	1.2921 × 10^4^	3.6584 × 10^4^	2.5310 × 10^3^	1.6783 × 10^4^	**3.0001 × 10^2^**	5.1741 × 10^4^
	Std	1.3218 × 10^4^	2.4281 × 10^3^	1.3179 × 10^4^	1.7331 × 10^2^	3.4541 × 10^3^	1.1725 × 10^4^	1.4810 × 10^3^	5.0877 × 10^3^	**9.7123 × 10^−3^**	1.3218 × 10^4^
F2	Ave	5.2064 × 10^2^	4.7463 × 10^2^	4.5043 × 10^2^	4.5474 × 10^2^	4.5774 × 10^2^	5.2249 × 10^2^	4.6097 × 10^2^	6.3774 × 10^2^	**4.3326 × 10^2^**	5.2064 × 10^2^
	Std	6.1177 × 10^1^	1.9339 × 10^1^	**1.1172 × 10^1^**	1.8372 × 10^1^	1.4083 × 10^1^	9.4574 × 10^1^	1.8465 × 10^1^	7.3974 × 10^1^	2.4467 × 10^1^	6.1177 × 10^1^
F3	Ave	6.3553 × 10^2^	6.2659 × 10^2^	6.0022 × 10^2^	6.1681 × 10^2^	6.0030 × 10^2^	6.3487 × 10^2^	6.0026 × 10^2^	6.4712 × 10^2^	**6.0002 × 10^2^**	6.3553 × 10^2^
	Std	1.4091 × 10^1^	8.3627 × 10^00^	2.6501 × 10^−1^	8.5315 × 10^00^	1.3367 × 10^−1^	1.4540 × 10^1^	3.5365 × 10^−1^	5.2523 × 10^00^	**1.4216 × 10^−2^**	1.4091 × 10^1^
F4	Ave	9.1426 × 10^2^	8.6379 × 10^2^	**8.3022 × 10^2^**	8.6099 × 10^2^	9.0083 × 10^2^	9.0913 × 10^2^	8.3895 × 10^2^	8.6598 × 10^2^	8.4091 × 10^2^	9.1426 × 10^2^
	Std	2.4271 × 10^1^	1.6192 × 10^1^	**5.7906 × 10^00^**	1.5133 × 10^1^	1.2476 × 10^1^	2.9378 × 10^1^	1.5089 × 10^1^	1.3646 × 10^1^	7.3521 × 10^00^	2.4271 × 10^1^
F5	Ave	3.4805 × 10^3^	1.6867 × 10^3^	9.5486 × 10^2^	1.6097 × 10^3^	**9.1204 × 10^2^**	2.1354 × 10^3^	9.4273 × 10^2^	1.9701 × 10^3^	9.5504 × 10^2^	3.4805 × 10^3^
	Std	1.1832 × 10^3^	5.2830 × 10^2^	5.4178 × 10^1^	5.8449 × 10^2^	**1.5834 × 10^1^**	6.2007 × 10^2^	9.9783 × 10^1^	2.8375 × 10^2^	1.2619 × 10^2^	1.1832 × 10^3^
F6	Ave	6.7343 × 10^6^	4.0575 × 10^3^	4.5583 × 10^3^	6.8227 × 10^3^	2.1947 × 10^4^	2.2464 × 10^6^	5.0954 × 10^3^	8.6269 × 10^6^	**1.8382 × 10^3^**	6.7343 × 10^6^
	Std	2.1364 × 10^7^	2.6614 × 10^3^	3.9619 × 10^3^	6.5496 × 10^3^	1.2565 × 10^4^	1.0841 × 10^7^	4.7841 × 10^3^	1.4019 × 10^7^	**1.4928 × 10^1^**	2.1364 × 10^7^
F7	Ave	2.1439 × 10^3^	2.1104 × 10^3^	2.0490 × 10^3^	2.1065 × 10^3^	2.0626 × 10^3^	2.1448 × 10^3^	**2.0397 × 10^3^**	2.1219 × 10^3^	2.0450 × 10^3^	2.1439 × 10^3^
	Std	3.9351 × 10^1^	4.1183 × 10^1^	1.2764 × 10^1^	4.2539 × 10^1^	1.0119 × 10^1^	6.4521 × 10^1^	1.4688 × 10^1^	1.8756 × 10^1^	**8.3161 × 10^00^**	3.9351 × 10^1^
F8	Ave	2.3162 × 10^3^	2.2545 × 10^3^	2.2246 × 10^3^	2.2707 × 10^3^	2.2320 × 10^3^	2.3225 × 10^3^	2.2266 × 10^3^	2.3124 × 10^3^	**2.2237 × 10^3^**	2.3162 × 10^3^
	Std	7.0339 × 10^1^	5.6698 × 10^1^	1.7678 × 10^00^	7.2525 × 10^1^	1.6628 × 10^00^	7.5851 × 10^1^	2.9678 × 10^00^	9.9243 × 10^1^	**6.1880 × 10^−1^**	7.0339 × 10^1^
F9	Ave	2.5103 × 10^3^	2.5084 × 10^3^	2.4808 × 10^3^	2.4819 × 10^3^	2.4817 × 10^3^	2.5130 × 10^3^	2.4808 × 10^3^	2.6215 × 10^3^	**2.4808 × 10^3^**	2.5103 × 10^3^
	Std	1.8049 × 10^1^	2.7289 × 10^1^	5.1383 × 10^−4^	9.7104 × 10^−1^	4.5808 × 10^−1^	2.6612 × 10^1^	2.7051 × 10^−3^	6.2254 × 10^1^	**1.8373 × 10^−10^**	1.8049 × 10^1^
F10	Ave	2.5105 × 10^3^	3.1750 × 10^3^	**2.4672 × 10^3^**	3.3529 × 10^3^	2.5321 × 10^3^	3.8757 × 10^3^	2.7651 × 10^3^	3.4923 × 10^3^	2.5317 × 10^3^	2.5105 × 10^3^
	Std	**8.1242 × 10^00^**	9.6968 × 10^2^	5.5746 × 10^1^	7.8661 × 10^2^	7.1316 × 10^1^	1.2404 × 10^3^	4.6722 × 10^2^	9.2838 × 10^2^	5.7457 × 10^1^	**8.1242 × 10^00^**
F11	Ave	3.7932 × 10^3^	**2.9199 × 10^3^**	2.9251 × 10^3^	2.9500 × 10^3^	2.9255 × 10^3^	3.1935 × 10^3^	2.9716 × 10^3^	4.8670 × 10^3^	2.9233 × 10^3^	3.7932 × 10^3^
	Std	5.9108 × 10^2^	1.1250 × 10^2^	7.5236 × 10^1^	6.4242 × 10^1^	4.5084 × 10^1^	5.1888 × 10^2^	2.4474 × 10^2^	1.0197 × 10^3^	**4.3018 × 10^1^**	5.9108 × 10^2^
F12	Ave	2.9993 × 10^3^	2.9883 × 10^3^	2.9521 × 10^3^	2.9733 × 10^3^	2.9850 × 10^3^	3.0260 × 10^3^	**2.9474 × 10^3^**	3.4346 × 10^3^	2.9554 × 10^3^	2.9993 × 10^3^
	Std	3.5843 × 10^1^	3.8639 × 10^1^	1.7406 × 10^1^	2.0666 × 10^1^	1.1858 × 10^1^	3.9810 × 10^1^	**1.0692 × 10^1^**	1.6066 × 10^2^	2.8107 × 10^1^	3.5843 × 10^1^

**Table 5:** Results for various algorithms for the CEC 2020 and CEC2022.

Statistical Results	CEC2017 d = 30 (+/=/-)	CEC2022 d = 10 (+/=/-)	CEC2022 d = 20 (+/=/-)
IAGWO	(27/0/3)	(12/0/0)	(12/0/0)
EWOA	(30/0/0)	(11/0/1)	(11/0/1)
VPPSO	(29/0/1)	(11/0/1)	(12/0/0)
L-SHADE	(26/0/4)	(10/0/2)	(11/0/1)
AOO	(27/0/3)	(12/0/0)	(12/0/0)
DBO	(30/0/0)	(11/0/1)	(11/0/1)
CPO	(30/0/0)	(12/0/0)	(12/0/0)
SBOA	(24/0/6)	(7/0/5)	(9/0/3)
ZOA	(30/0/0)	(12/0/0)	(12/0/0)

**Table 6:** Friedman mean-rank test results.

Suites	CEC2017		CEC2022			
Dimensions	30		10		20	
Algorithms	M.R	T.R	M.R	T.R	M.R	T.R
IAGWO	5.87	6	5.58	5	5.75	7
EWOA	8.67	9	8.42	9	8.83	9
VPPSO	6.10	7	5.83	6	5.50	6
L-SHADE	2.60	2	2.67	2	2.50	2
AOO	4.83	4	6.25	7	5.08	4
DBO	5.27	5	4.08	4	5.42	5
CPO	8.50	8	8.33	8	8.50	8
SBOA	2.87	3	3.42	3	2.92	3
ZOA	8.93	10	8.92	10	8.92	10
SZOA	**1.37**	**1**	**1.50**	**1**	**1.58**	**1**

**Table 7:** Parameters of distributed power sources in the microgrid.

Power Type	Minimum Power (kW)	Maximum Power (kW)	Operating Cost ($·kW^−1^)	Fuel Cost ($·kW^−1^)
PV	0	35	0.0096	0
WT	0	45	0.45	0
FC	0	40	0.02933	0.2435
MT	0	40	0.0419	0.4090
GS	0	40	0.1258	0.6031
BT	−40	40	0.055	0

**Table 8:** Cost results obtained by different algorithms.

Algorithm	Max	Min	Mean	Std	Rank
IAGWO	IAGWO	10,074.73	6260.32	7087.74	702.62
EWOA	EWOA	8388.86	6413.31	7166.66	498.01
VPPSO	VPPSO	8814.37	5534.11	6967.01	691.59
L-SHADE	L-SHADE	8668.87	5717.85	6880.38	606.73
AOO	AOO	8137.78	5828.87	7028.82	599.02
CPO	CPO	**7715.83**	5539.75	6943.15	661.90
DBO	DBO	9123.10	5806.72	6993.85	576.53
SBOA	SBOA	7858.13	5758.07	6946.19	647.47
ZOA	ZOA	7721.94	6171.33	6957.48	531.31
SZOA	SZOA	8291.60	**5165.96**	**6853.07**	**448.53**

## Data Availability

All data are included in the manuscript.

## Appendix A

### Appendix A.1. Abbreviation Comparison Table

Grey Wolf Optimization	IAGWO
Enhanced Whale Optimization Algorithm	EWOA
Velocity Pausing Particle Swarm Optimization	VPPSO
Improving the Search Performance of SHADE	L-SHADE
Animated Oat Optimization	AOO
Crested Porcupine Optimizer	CPO
Dung Beetle Optimization Algorithm	DBO
Zebra Optimization Algorithm	ZOA
Synergistic Zebra Optimization	SZOA
Photovoltaic	PV
Wind Turbine	WT
Fuel Cells	FC
Small Internal Combustion Engines	GS
Batteries	BT
Micro Gas Turbines	MT
Main-Grid Interaction Model	GRID
Fuel Cost	$F_{\mathrm{Fuel}\;}$
Operating Cost	$F_{Op}$
Pollutant Treatment Cost	$F_{Poll}$
Grid Interaction Cost	$F_{\mathrm{Grid}\;}$
Power Imbalance Penalty Cost	$F_{Pen}$
State-of-Charge	SOC
Optimal Scheduling	OS
Energy Management System	EMS

### Appendix A.2. The p-Value on Test Suites

Results: p-value for CEC2017 (d = 30).

**Function**	**IAGWO**	**EWOA**	**VPPSO**	**L-SHADE**	**AOO**	**CPO**	**DBO**	**SBOA**	**ZOA**
F1	3.0199 × 10^−11^	3.0199 × 10^−11^	**0.06567126**	1.35943 × 10^−7^	3.33839 × 10^−11^	3.01986 × 10^−11^	3.01986 × 10^−11^	1.4294 × 10^−8^	3.01986 × 10^−11^
F2	3.0199 × 10^−11^	3.0199 × 10^−11^	3.0199 × 10^−11^	3.01986 × 10^−11^	3.01986 × 10^−11^	3.01986 × 10^−11^	3.01986 × 10^−11^	3.0199 × 10^−11^	3.01986 × 10^−11^
F3	3.0199 × 10^−11^	3.0199 × 10^−11^	3.0199 × 10^−11^	3.01986 × 10^−11^	3.01986 × 10^−11^	3.01986 × 10^−11^	3.01986 × 10^−11^	3.0199 × 10^−11^	3.01986 × 10^−11^
F4	2.2273 × 10^−9^	3.0199 × 10^−11^	3.1967 × 10^−9^	2.83145 × 10^−8^	2.67842 × 10^−6^	3.80526 × 10^−7^	4.97517 × 10^−11^	3.3681 × 10^−5^	3.01986 × 10^−11^
F5	7.7725 × 10^−9^	3.0199 × 10^−11^	1.1023 × 10^−8^	3.01986 × 10^−11^	0.000132495	3.01986 × 10^−11^	3.33839 × 10^−11^	0.00463712	3.01986 × 10^−11^
F6	3.0199 × 10^−11^	3.0199 × 10^−11^	3.0199 × 10^−11^	3.01986 × 10^−11^	3.01986 × 10^−11^	3.01986 × 10^−11^	3.01986 × 10^−11^	3.0199 × 10^−11^	3.01986 × 10^−11^
F7	6.1210 × 10^−10^	3.0199 × 10^−11^	1.287 × 10^−9^	3.01986 × 10^−11^	5.46203 × 10^−6^	3.01986 × 10^−11^	3.01986 × 10^−11^	**0.18089953**	3.01986 × 10^−11^
F8	3.8053 × 10^−7^	3.0199 × 10^−11^	1.0666 × 10^−7^	5.57265 × 10^−10^	7.22083 × 10^−6^	3.01986 × 10^−11^	3.01986 × 10^−11^	0.00100353	7.38908 × 10^−11^
F9	3.0199 × 10^−11^	3.0199 × 10^−11^	4.5043 × 10^−11^	3.01986 × 10^−11^	7.38908 × 10^−11^	0.002754848	3.01986 × 10^−11^	0.00215664	3.01986 × 10^−11^
F10	**7.7272 × 10^−2^**	3.0199 × 10^−11^	6.7362 × 10^−6^	8.84109 × 10^−7^	0.00037704	3.01986 × 10^−11^	1.32885 × 10^−10^	**0.11536236**	2.1544 × 10^−10^
F11	6.6955 × 10^−11^	3.0199 × 10^−11^	3.0199 × 10^−11^	1.20233 × 10^−8^	1.77691 × 10^−10^	3.01986 × 10^−11^	3.01986 × 10^−11^	2.3897 × 10^−8^	3.01986 × 10^−11^
F12	3.0199 × 10^−11^	3.0199 × 10^−11^	3.0199 × 10^−11^	3.01986 × 10^−11^	3.01986 × 10^−11^	4.07716 × 10^−11^	3.01986 × 10^−11^	5.4941 × 10^−11^	3.01986 × 10^−11^
F13	4.9752 × 10^−11^	3.0199 × 10^−11^	3.0199 × 10^−11^	3.01986 × 10^−11^	3.01986 × 10^−11^	3.33839 × 10^−11^	3.01986 × 10^−11^	4.0772 × 10^−11^	3.01986 × 10^−11^
F14	3.0199 × 10^−11^	3.0199 × 10^−11^	3.0199 × 10^−11^	3.01986 × 10^−11^	3.01986 × 10^−11^	3.01986 × 10^−11^	3.01986 × 10^−11^	3.0199 × 10^−11^	3.01986 × 10^−11^
F15	6.6955 × 10^−11^	3.0199 × 10^−11^	3.0199 × 10^−11^	3.01986 × 10^−11^	3.01986 × 10^−11^	3.01986 × 10^−11^	3.01986 × 10^−11^	3.0199 × 10^−11^	3.01986 × 10^−11^
F16	8.9934 × 10^−11^	3.6897 × 10^−11^	4.5726 × 10^−9^	3.25554 × 10^−7^	3.32415 × 10^−6^	6.69552 × 10^−11^	3.01986 × 10^−11^	**0.88830284**	3.01986 × 10^−11^
F17	3.3520 × 10^−8^	3.0199 × 10^−11^	5.0912 × 10^−6^	1.4918 × 10^−6^	5.26501 × 10^−5^	0.000104066	4.50432 × 10^−11^	**0.53951032**	2.60985 × 10^−10^
F18	3.0199 × 10^−11^	3.0199 × 10^−11^	3.0199 × 10^−11^	3.01986 × 10^−11^	3.01986 × 10^−11^	3.01986 × 10^−11^	3.01986 × 10^−11^	3.0199 × 10^−11^	3.01986 × 10^−11^
F19	3.0199 × 10^−11^	3.0199 × 10^−11^	3.0199 × 10^−11^	3.01986 × 10^−11^	3.01986 × 10^−11^	3.01986 × 10^−11^	3.01986 × 10^−11^	3.3384 × 10^−11^	3.01986 × 10^−11^
F20	1.0105 × 10^−8^	4.5043 × 10^−11^	5.0723 × 10^−10^	1.8731 × 10^−7^	1.55808 × 10^−8^	1.15665 × 10^−7^	5.49405 × 10^−11^	**0.23398892**	3.19674 × 10^−9^
F21	1.6947 × 10^−9^	3.0199 × 10^−11^	7.043 × 10^−7^	1.72903 × 10^−6^	0.008684371	3.01986 × 10^−11^	3.01986 × 10^−11^	1.1674 × 10^−5^	3.01986 × 10^−11^
F22	4.6159 × 10^−10^	8.9934 × 10^−11^	0.00137033	1.28604 × 10^−6^	6.51827 × 10^−9^	1.07018 × 10^−9^	1.09367 × 10^−10^	0.00065486	9.91863 × 10^−11^
F23	3.0199 × 10^−11^	3.0199 × 10^−11^	2.4386 × 10^−9^	7.38029 × 10^−10^	1.42942 × 10^−8^	3.01986 × 10^−11^	3.01986 × 10^−11^	0.01836796	3.01986 × 10^−11^
F24	3.0199 × 10^−11^	6.6955 × 10^−11^	0.00275485	**0.16686617**	0.019883076	2.37147 × 10^−10^	3.01986 × 10^−11^	5.9673 × 10^−9^	3.01986 × 10^−11^
F25	5.4941 × 10^−11^	3.0199 × 10^−11^	9.7555 × 10^−10^	4.31061 × 10^−8^	0.00033679	2.57212 × 10^−7^	5.49405 × 10^−11^	8.2919 × 10^−6^	3.01986 × 10^−11^
F26	**5.9428 × 10^−2^**	3.0199 × 10^−11^	8.2919 × 10^−6^	3.35195 × 10^−8^	0.000654865	3.83494 × 10^−6^	3.4742 × 10^−10^	0.01122776	3.01986 × 10^−11^
F27	4.8413 × 10^−2^	1.3289 × 10^−10^	6.6955 × 10^−11^	5.49405 × 10^−11^	4.61591 × 10^−10^	3.01986 × 10^−11^	3.33839 × 10^−11^	0.03265094	3.01986 × 10^−11^
F28	3.0199 × 10^−11^	3.0199 × 10^−11^	3.0199 × 10^−11^	3.01986 × 10^−11^	1.32885 × 10^−10^	3.01986 × 10^−11^	3.01986 × 10^−11^	1.2057 × 10^−10^	3.01986 × 10^−11^
F29	9.0632 × 10^−8^	3.6897 × 10^−11^	3.3384 × 10^−11^	3.33839 × 10^−11^	2.37147 × 10^−10^	5.49405 × 10^−11^	3.33839 × 10^−11^	**0.10232627**	3.01986 × 10^−11^
F30	**2.1156 × 10^−1^**	3.0199 × 10^−11^	3.0199 × 10^−11^	3.01986 × 10^−11^	3.01986 × 10^−11^	3.01986 × 10^−11^	3.01986 × 10^−11^	8.1527 × 10^−11^	3.01986 × 10^−11^

Results: p-value for CEC2022 (d = 10).

**Function**	**IAGWO**	**EWOA**	**VPPSO**	**L-SHADE**	**AOO**	**CPO**	**DBO**	**SBOA**	**ZOA**
F1	1.0149 × 10^−11^	1.0149 × 10^−11^	1.0149 × 10^−11^	1.0149 × 10^−11^	1.0149 × 10^−11^	1.0149 × 10^−11^	1.0149 × 10^−11^	1.0149 × 10^−11^	1.0149 × 10^−11^
F2	1.1089 × 10^−8^	2.1362 × 10^−11^	3.1229 × 10^−8^	5.18008 × 10^−8^	6.53122 × 10^−9^	8.128 × 10^−6^	3.91896 × 10^−11^	2.1078 × 10^−9^	1.29084 × 10^−10^
F3	3.0199 × 10^−11^	3.0199 × 10^−11^	3.0199 × 10^−11^	3.01986 × 10^−11^	3.01986 × 10^−11^	1.55808 × 10^−8^	3.01986 × 10^−11^	**0.07978165**	3.01986 × 10^−11^
F4	2.4386 × 10^−9^	4.5043 × 10^−11^	7.3803 × 10^−10^	3.01986 × 10^−11^	5.18568 × 10^−7^	3.68973 × 10^−11^	3.01986 × 10^−11^	0.00123618	1.28704 × 10^−9^
F5	2.4967 × 10^−11^	1.087 × 10^−11^	2.252 × 10^−11^	1.087 × 10^−11^	5.64044 × 10^−9^	5.9788 × 10^−6^	1.087 × 10^−11^	9.3414 × 10^−7^	1.087 × 10^−11^
F6	3.0199 × 10^−11^	3.0199 × 10^−11^	3.0199 × 10^−11^	3.01986 × 10^−11^	3.01986 × 10^−11^	3.01986 × 10^−11^	3.01986 × 10^−11^	3.0199 × 10^−11^	3.01986 × 10^−11^
F7	4.0330 × 10^−3^	4.9752 × 10^−11^	4.0772 × 10^−11^	4.97517 × 10^−11^	1.46431 × 10^−10^	0.000140669	4.97517 × 10^−11^	**0.12597019**	3.33839 × 10^−11^
F8	2.8790 × 10^−6^	3.0199 × 10^−11^	2.2273 × 10^−9^	6.12104 × 10^−10^	2.38974 × 10^−8^	0.01383162	2.0338 × 10^−9^	0.03778202	1.32885 × 10^−10^
F9	5.1138 × 10^−3^	2.3638 × 10^−12^	2.3638 × 10^−12^	2.36384 × 10^−12^	2.36384 × 10^−12^	2.36384 × 10^−12^	2.41705 × 10^−7^	**0.63240796**	2.36384 × 10^−12^
F10	4.6390 × 10^−5^	**0.18576686**	0.00065486	0.000178356	0.000654865	**0.090490361**	0.000620265	**0.97051605**	6.76501 × 10^−5^
F11	6.7716 × 10^−6^	3.4718 × 10^−5^	3.9409 × 10^−7^	5.12356 × 10^−5^	2.28809 × 10^−6^	5.12356 × 10^−5^	2.33433 × 10^−5^	3.0705 × 10^−6^	8.44688 × 10^−8^
F12	6.2434 × 10^−5^	2.9249 × 10^−7^	**0.36271509**	0.007226745	0.029035155	2.73787 × 10^−8^	1.16054 × 10^−8^	**0.06304368**	2.87907 × 10^−11^

Results: p-value for CEC2022 (d = 20).

**Function**	**IAGWO**	**EWOA**	**VPPSO**	**L-SHADE**	**AOO**	**CPO**	**DBO**	**SBOA**	**ZOA**
F1	3.0199 × 10^−11^	3.0199 × 10^−11^	3.0199 × 10^−11^	3.01986 × 10^−11^	3.01986 × 10^−11^	3.01986 × 10^−11^	3.01986 × 10^−11^	3.0199 × 10^−11^	3.01986 × 10^−11^
F2	5.4941 × 10^−11^	8.1527 × 10^−11^	3.8249 × 10^−9^	5.53286 × 10^−8^	1.25408 × 10^−7^	9.7555 × 10^−10^	3.4742 × 10^−10^	4.1825 × 10^−9^	3.01986 × 10^−11^
F3	3.0199 × 10^−11^	3.0199 × 10^−11^	3.0199 × 10^−11^	3.01986 × 10^−11^	3.01986 × 10^−11^	3.01986 × 10^−11^	3.01986 × 10^−11^	2.5721 × 10^−7^	3.01986 × 10^−11^
F4	4.9980 × 10^−9^	3.0199 × 10^−11^	2.1959 × 10^−7^	4.57257 × 10^−9^	0.001679756	3.01986 × 10^−11^	6.06576 × 10^−11^	**0.7282653**	2.60985 × 10^−10^
F5	1.9568 × 10^−10^	4.5043 × 10^−11^	1.5465 × 10^−9^	1.09367 × 10^−10^	3.82489 × 10^−9^	0.004427193	1.95678 × 10^−10^	1.4298 × 10^−5^	8.99341 × 10^−11^
F6	4.9752 × 10^−11^	3.0199 × 10^−11^	4.0772 × 10^−11^	3.68973 × 10^−11^	3.68973 × 10^−11^	3.01986 × 10^−11^	3.01986 × 10^−11^	3.0199 × 10^−11^	3.01986 × 10^−11^
F7	6.5486 × 10^−4^	4.0772 × 10^−11^	1.85 × 10^−8^	4.07716 × 10^−11^	3.52006 × 10^−7^	1.25408 × 10^−7^	2.87158 × 10^−10^	0.02920541	4.07716 × 10^−11^
F8	1.5638 × 10^−2^	1.4643 × 10^−10^	2.0338 × 10^−9^	1.69795 × 10^−8^	3.4742 × 10^−10^	5.57265 × 10^−10^	2.43863 × 10^−9^	9.5332 × 10^−7^	1.46431 × 10^−10^
F9	5.5727 × 10^−10^	3.0199 × 10^−11^	3.0199 × 10^−11^	3.01986 × 10^−11^	3.01986 × 10^−11^	3.01986 × 10^−11^	3.01986 × 10^−11^	3.0199 × 10^−11^	3.01986 × 10^−11^
F10	4.1127 × 10^−7^	**0.17145005**	0.00065486	0.010762613	2.37682 × 10^−7^	**0.491782957**	0.000356384	**0.73939882**	6.2828 × 10^−6^
F11	1.5846 × 10^−4^	5.4941 × 10^−11^	5.462 × 10^−6^	7.73869 × 10^−6^	5.97056 × 10^−5^	0.000421751	2.22727 × 10^−9^	0.00031821	3.01986 × 10^−11^
F12	7.9590 × 10^−3^	1.3111 × 10^−8^	2.879 × 10^−6^	6.04595 × 10^−7^	3.15727 × 10^−5^	1.61323 × 10^−10^	6.12104 × 10^−10^	**0.0724456**	3.01986 × 10^−11^
